# C-Terminal Peptide Modifications Reveal Direct and Indirect Roles of Hedgehog Morphogen Cholesteroylation

**DOI:** 10.3389/fcell.2020.615698

**Published:** 2021-01-12

**Authors:** Dominique Manikowski, Philipp Kastl, Sabine Schürmann, Kristina Ehring, Georg Steffes, Petra Jakobs, Kay Grobe

**Affiliations:** ^1^Institute of Physiological Chemistry and Pathobiochemistry and the Cells-in-Motion Cluster of Excellence (EXC1003-CiM), University of Münster, Münster, Germany; ^2^Institute of Neuro- and Behavioral Biology, University of Münster, Münster, Germany

**Keywords:** hedgehog, Sonic hedgehog, *Drosophila*, proteolysis, morphogen, signaling

## Abstract

Hedgehog (Hh) morphogens are involved in embryonic development and stem cell biology and, if misregulated, can contribute to cancer. One important post-translational modification with profound impact on Hh biofunction is its C-terminal cholesteroylation during biosynthesis. The current hypothesis is that the cholesterol moiety is a decisive factor in Hh association with the outer plasma membrane leaflet of producing cells, cell-surface Hh multimerization, and its transport and signaling. Yet, it is not decided whether the cholesterol moiety is directly involved in all of these processes, because their functional interdependency raises the alternative possibility that the cholesterol initiates early processes directly and that these processes can then steer later stages of Hh signaling independent of the lipid. We generated variants of the C-terminal Hh peptide and observed that these cholesteroylated peptides variably impaired several post-translational processes in producing cells and Hh biofunction in *Drosophila melanogaster* eye and wing development. We also found that substantial Hh amounts separated from cholesteroylated peptide tags *in vitro* and *in vivo* and that tagged and untagged Hh variants lacking their C-cholesterol moieties remained bioactive. Our approach thus confirms that Hh cholesteroylation is essential during the early steps of Hh production and maturation but also suggests that it is dispensable for Hh signal reception at receiving cells.

## Introduction

Cell fate determination during development is controlled by morphogen gradients. In vertebrates, three closely related Hedgehog morphogens [Sonic hedgehog (Shh), Indian hedgehog, and Desert hedgehog] directly and concentration dependently regulate cell differentiation, cell proliferation, and tissue polarity during embryogenesis. In *Drosophila melanogaster*, a single Hedgehog (Hh) ortholog determines embryonic segment polarity and regulates growth and patterning of wings and eyes. Hh biosynthesis and signaling are highly conserved from flies to humans (Ingham and Placzek, 2006). Its production begins with the cleavage of a signal sequence from a 45 kDa precursor molecule during endoplasmic reticulum (ER) export, followed by coupled autocatalytic cleavage and cholesteroylation of the 19 kDa N-terminal signaling domain by the autocatalytic cholesterol transferase domain (Porter et al., 1996b). Subsequently, the C-terminally cholesterol-modified signaling domain is N-terminally palmitoylated, resulting in dual-lipidated Hh that constitutes the fully active morphogen (Jacob and Lum, 2007). In contrast to the N-palmitate, which directly (Tukachinsky et al., 2016; Qi et al., 2018) or indirectly (Ohlig et al., 2011; Kastl et al., 2018; Schürmann et al., 2018) controls Hh biofunction, artificially truncated Hh cDNA lacking the C-terminal domain is translated into non-cholesteroylated yet bioactive 19 kDa HhN *in vitro* (Zeng et al., 2001; Dawber et al., 2005) and *in vivo* (Porter et al., 1996a; Lewis et al., 2001; Li et al., 2006).

Yet, despite its non-essential role in Hh signaling, the cholesterol moiety is known to contribute to the spatiotemporal control of Hh function at several points during Hh production. The first role of the C-terminally linked cholesterol is to associate nascent lipidated Hhs with the cell membrane of producing cells to prevent their uncontrolled secretion and instead to allow for regulated long-distance Hh transport in vertebrates and invertebrates (Dawber et al., 2005; Callejo et al., 2006; Gallet et al., 2006; Huang et al., 2007). Using cell-surface-associated heparan sulfate (HS) proteoglycans as scaffolds, lipidated Hhs then form large multimers (Vyas et al., 2008). An alternative approach to explain Hh clustering was via cholesterol-dependent Hh assembly into micelles (Zeng et al., 2001), their insertion into lipoprotein particles (Panakova et al., 2005) or exosomes to mediate Hh transport (Gradilla et al., 2014; Vyas et al., 2014), or that C-cholesterol aids Hh transport through or on extended filopodia-like structures called cytonemes (Torroja et al., 2004; Chen et al., 2017; Gonzalez-Mendez et al., 2017).

Another suggested transporter of the vertebrate Hh ortholog Shh is the secreted glycoprotein Scube2 (Signal peptide, CUB-EGF-like domain-containing protein 2). Scube2 is a member of the *you* class mutants in zebrafish and is essential for regulated Shh signaling *in vitro* and *in vivo* (Kawakami et al., 2005; Woods and Talbot, 2005; Hollway et al., 2006; Tsai et al., 2009). Together with a Hh-specific 12-pass transmembrane protein called Dispatched (Disp) (Burke et al., 1999), Scube2 increases Shh release from the surface of producing cells by about 10-fold (Creanga et al., 2012). This activity depends on the Scube2 CUB (C1r/C1s, Uegf, and Bmp1) domain, because Scube2^Δ*CUB*^, a truncation mutant lacking this domain, is inactive (Creanga et al., 2012; Tukachinsky et al., 2012; Jakobs et al., 2014). One way to explain this is that Disp extracts Hh cholesterol from the cell membrane for subsequent Scube2 CUB domain-mediated transport to receiving cells (Tukachinsky et al., 2012). However, CUB domains derive their name from the complement subcomponents C1r/C1s, sea urchin protein with EGF-like domains (UEGF), and bone morphogenetic protein 1 (BMP1) and contribute to protease activities in these proteins (Gaboriaud et al., 2011), possibly by inducing structural substrate changes to boost their turnover (Bourhis et al., 2013). An alternative possibility therefore is that cholesterol tethers Hh to the plasma membrane only until the morphogen is released by Scube2 CUB domain-regulated proteolytic processing (Farshi et al., 2011; Ohlig et al., 2011, 2012; Jakobs et al., 2014, 2016, 2017; Ortmann et al., 2015). Depending on which of these mechanistic models of Scube2 function is correct, cholesterol might (Qian et al., 2019) or might not (Gong et al., 2018) contribute to Hh binding to its receptor Patched (Ptc) at the surface of receiving cells.

In this work, we analyzed the functional consequences of C-terminal peptide tags on cholesterol-regulated Hh multimerization, release, and signaling. In the past, a similar approach has been used to target the most N-terminal palmitoylated Hh peptide: Just like the Hh cholesterol moiety, Hh palmitoylation was previously thought to act as a direct positive regulator of Hh biofunction. However, the insertion of hemagglutinin (HA) tags into the palmitoylated N-terminal peptide not only rendered palmitoylated Hh inactive but also suppressed endogenous Hh function during wing development in *Drosophila melanogaster* (Kastl et al., 2018; Schürmann et al., 2018). Notably, additional palmitate removal restored wing and eye development, suggesting that palmitate acts as a negative regulator of Hh biofunction as long as it anchors Hh clusters to the surface of producing cells. The rationale for our C-terminal tagging strategy presented in this work therefore was to test whether the Hh cholesterol moiety functions in a similar way. We found that the sequence and length of the C-terminal peptide affected Hh biofunction in wing and eye development, albeit less than the N-terminal Hh peptide. We also found that insertional mutagenesis attenuated dominant-negative suppression of Hh-dependent wing and restored eye developmental defects caused by N-terminal peptide modifications *in vivo*, probably by affecting physical interactions between both proteins at the cell surface. Finally, our strategy revealed extensive proteolytic Hh separation from cholesteroylated tags *in vitro* and *in vivo*, yet Hh variants lacking C-cholesterol remained signaling-active. These data suggest that precise, spatiotemporally controlled Hh signaling on producing cells requires and is initiated by the cholesterol moiety, yet its role at later signaling steps becomes increasingly indirect. This challenges current *in vitro* derived concepts of Hh transport and signaling in cholesteroylated form.

## Materials and Methods

### Fly Lines

The following GAL4 driver lines were used: En-GAL4e16E (en >): P(en2.4-GAL4)e16E, FlyBase ID FBrf0098595; en(2)-GAL4 [en(2) >]: w[1118]; P{y[+t7.7]w+mC]=GMR94D09-GAL4}attP2, Bloomington stock #48011; Hh-GAL4 (hh >): w[*]; P{w[+mC]=GAL4}hh[Gal4]/TM6B,Tb[1], Bloomington stock #67046; GMR-GAL4 (GMR >): GMR17G12 (GMR45433-GAL4): P[y( + t7.7)w( + mC) = GMR17G12-GAL4]*attP2*, Bloomington stock #45433 (discontinued but available from our lab), SGS-GAL4 (SGS >): w(1118); P[w( + mC) = Sgs3-GAL4.PD]TP1, Bloomington stock #6870. Other flies used were *hh^*bar*3^*, FlyBase ID FBal0031487 and *hh*^*AC*^: *ry(506) hh(AC)/TM3, Sb(1)*, Bloomington stock #1749. Hh and all Hh variant constructs were inserted into the same 51C1 landing site (BestGene) by using germline-specific PhiC31 integrase (Bateman et al., 2006). Correct protein processing and secretion of *pUAST-attB-hh* constructs was confirmed by *Drosophila* S2 cell expression, and their expression under the control of Ptc-Gal4 arrested fly development at the same embryo-L2 stages. This indicated similar bioactivity of Hh and Hh variants (not shown). *yw; Hh^*GFP BAC*^/Hh^*GFP BAC*^* flies were kindly provided by Thomas Kornberg, University of California, San Francisco, United States. *yw; Hh^*GFP*^/Hh^*GFP*^; MKRS/Tm6B*, *yw; If/GVDI; ^*C*85S^Hh^*GFP*^/Tm6B*, and y*w; HhN^*GFP*^/HhN^*GFP*^; MKRS/TM6B* flies were kindly provided by Isabel Guerrero, Universidad Autónoma de Madrid, Spain. PtcLacZ reporter flies were kindly provided by Jianhang Jia, Markey Cancer Center, and Department of Molecular and Cellular Biochemistry, University of Kentucky College of Medicine, Lexington, United States. The *w^–^; P(en2.4-GAL4)e16E.UAS-Cherry; TM2/TM6B* driver/reporter line was kindly provided by Christian Klämbt, Institute of Neuro- and Behavioral Biology, University of Münster, Germany.

### Eye Development Analysis

Hh transgene expression in the morphogenetic furrow of the eye disc was conducted by crossing the following fly lines: *UAS-hh^∗^/CyO^*WeeP*^; hh^*AC*^/Tm6B*, and *GMR-GAL4/GMR-GAL4; hh^*bar*3^/hh^*bar*3^* and kept at 18°C or 25°C. Ommatidia number of the resulting *UAS-Hh^∗^/GMR-GAL4; hh^*bar*3^/hh^*AC*^* flies were analyzed with a Nikon SMZ25 microscope. *white*^1118^ flies served as positive controls and; + / + ; *hh^*bar*3^/hh^*AC*^* flies served as negative controls.

### Wing Development Analysis

Hh transgene expression in the posterior imaginal wing disc was obtained by crossing *En-GAL4e16E* (en > *): P(en2.4-GAL4)e16E or w^–^; P(en2.4-GAL4)e16E.UAS-Cherry; TM2/TM6B or Hh-GAL4* (hh > ): w[*]; P{w[+mC]=GAL4}hh[Gal4]/TM6B,Tb[1] with UAS-hh^∗^ variants and kept at 25°C. The resulting wings of the en-Gal4/hh^∗^ flies and of; hh*/+;hh-Gal4/+ flies were analyzed with a Motic SMZ-168 microscope equipped with a moticam 2,300. The L3–L4 intervein areas and the L2–L3 intervein areas were measured with ImageJ and the ratios thereof calculated.

### Wing Disc Analysis

At least five wing discs per genotype/experiment were fixed with 4% PFA for 1 h at 4°C, permeabilized with 1% Triton X-100 for 30 min at room temperature. Samples were stained with rabbit anti-α-galactosidase IgG antibodies (Cappel, MP Biomedicals) followed by Alexa488-conjugated goat-α-rabbit IgG antibodies (Jackson ImmunoResearch). The posterior compartment was intrinsically labeled with en > UAS-Cherry. Images were taken on a LSM 700 Zeiss confocal microscope with ZEN software. Maximum intensity projections are shown. Plot profiles were generated with ImageJ and GraphPad Prism.

### Size Exclusion Chromatography (SEC) of Larval Homogenates

En(2)-Gal4 (en(2) >): w[1118]; P{y[+t7.7]w+mC]=GMR94D09-GAL4}attP2 flies were crossed with UAS-hh^∗^ flies and kept at 25°C. Approximately 50 mg of whole 3rd-instar larvae or 3rd-instar imaginal discs were homogenized and suspended in phosphate-buffered saline (PBS) supplemented with Protease Inhibitor Cocktail (Roche, Basel) either with or without 0.1% Triton X-100. Soluble proteins were cleared from remaining cellular debris and larval fat by centrifugation. The cleared fraction was microfiltered and subjected to SEC analysis by using an Äkta protein purifier (GE Healthcare) on a Superdex 200 10/300 GL column (Pharmacia) equilibrated with PBS at 4°C. Eluted fractions were trichloroacetic acid (TCA) precipitated and analyzed by SDS-PAGE and Western blot using rabbit-α-dHh antibodies (Santa Cruz), rabbit α-GFP (Sigma) or goat α-GFP (Rockland) antibodies followed by HRP-conjugated secondary antibodies. Enhanced chemiluminescence (ECL) signals were detected and quantified by using ImageJ. The most abundant protein expression was set to 100%. Plot profiles were generated by using MS Excel. As control experiments, homogenates of SGS-Gal4 (SGS >) or en(2)-Gal4-driven Hh^*GFP*^ expressing larvae were directly lyzed in reducing SDS sample buffer and subjected to SDS-PAGE and Western blot analysis using rabbit α-GFP (Sigma) antibodies followed by HRP conjugated secondary antibodies and ECL detection.

### Cloning and Expression of Recombinant Drosophila Hh Proteins

*Hh* cDNA (nucleotides 1–1,416, corresponding to amino acids 1–471 of *D. melanogaster* Hh) and *HhN* cDNA (nucleotides 1–771, corresponding to amino acids 1–257) were inserted into pENTR, sequenced, and cloned into pUAST-attB for protein expression in S2 cells or the generation of genetically modified flies. Tags were introduced by QuikChange Lightning site-directed mutagenesis (Agilent). Primer sequences can be provided upon request. S2 cells were cultured in Schneider’s medium (Invitrogen) supplemented with 10% fetal calf serum (FCS) and 100 μg/ml penicillin/streptomycin. S2 cells were transfected via Effectene (Qiagen) with constructs encoding ^*C*85S^HhN, Hh, or its C-terminal variant forms, together with a vector encoding *actin-GAL4*, and cultured for 48 h in Schneider’s medium before cells were fixed with 4% PFA for 10 min and stained with a rabbit-α-dHh antibody (Santa Cruz) followed by a Cy3-conjugated donkey α-rabbit IgG antibody (Jackson ImmunoResearch). Images were taken on a LSM 700 Zeiss confocal microscope with ZEN software. Maximum intensity projections are shown.

### Cloning and Expression of Recombinant Murine Shh Proteins

*Shh* cDNA (nucleotides 1–1,314, corresponding to amino acids 1–438 of *Mus musculus* Shh) and *ShhN* cDNA (nucleotides 1–594, corresponding to amino acids 1–198) were inserted into pcDNA3.1 (Invitrogen) or into pIRES for bicistronic expression of Shh and the hedgehog acyltransferase (Hhat) for efficient Shh palmitoylation. The insertion of the HA and StrepII tag was achieved by PCR using primers carrying the desired tag sequences. Primer sequences can be provided upon request. Shh^*GFP*^ was generated according to the published Hh^*GFP*^ fly (Torroja et al., 2004). In brief, GFP was amplified by PCR using *yw; Hh^*GFP*^/Hh^*GFP*^; MKRS/Tm6B* flies as template and cloned into pcDNA3.1 or pIRES carrying Shh^*K*195H, S196V^, where a *Pml*I cleavage site was introduced to insert the GFP tag.

### SEC of Soluble Shh

Bosc23 cells were cultured in DMEM supplemented with 10% FCS and 100 μg/ml penicillin/streptomycin. Cells were transfected with pIRES encoding Shh or its C-terminally variants and Hhat by using PolyFect (Qiagen). Cells were grown for 2 days, and the supernatant was centrifuged for cell debris removal. The cleared medium containing soluble Shh was microfiltered and subjected to SEC analysis by using an Äkta protein purifier (GE Healthcare) on a Superdex 200 10/300 GL column (GE Healthcare) equilibrated with PBS at 4°C. Eluted fractions were TCA precipitated and analyzed by SDS-PAGE and Western blot by using α-Shh (R&D systems) or α-StrepII antibodies. Signals were quantified by using ImageJ. Total eluted protein was set to 100% and ratios of monomeric (< 80 kDa) versus multimeric Shh^∗^ (> 80 kDa) were calculated.

### Tandem Affinity Chromatography of Soluble Shh

To determine HS-binding properties of C-terminally tagged Shh, the supernatant of transfected Bosc23 cells was subjected to affinity chromatography (Äkta Protein Purifier) using a tandem arrangement of low-sulfated *Drosophila melanogaster* HS coupled to NHS-activated sepharose columns and highly sulfated heparin sepharose columns (Pharmacia) at 4°C. In this arrangement, proteins were applied in the absence of salt to the HS-coupled column and the flow-through was directly applied to heparin sepharose. After washing, columns were separated and the bound material eluted with a linear NaCl gradient from 0 to 1.5 M in 0.1 M sodium acetate buffer (pH 6.0). Fractions were TCA precipitated, and eluted proteins were analyzed by SDS-PAGE and Western blots stained with α-Shh antibodies (R&D systems). HS binding was calculated as follows: detected HS-bound Shh/total Shh (= HS-bound Shh^∗^ + heparin-bound Shh^∗^).

### Reverse-Phase High-Performance Liquid Chromatography (HPLC)

Bosc23 cells were transfected with expression plasmids for unlipidated ^*C*25A^ShhN control protein and cholesteroylated (yet non-palmitoylated) ^*C*25S^Shh and C-terminally tagged, unpalmitoylated ^*C*25A^Shh^*HA*^. Two days post-transfection, cells were lysed in radioimmunoprecipitation assay buffer containing complete protease inhibitor cocktail (Roche, Basel, Switzerland) on ice, ultracentrifuged at 40,000 × g for 1 h, and the soluble whole-cell extract acetone-precipitated. Protein precipitates were resuspended in 35 μl of (1,1,1,3,3,3)-hexafluoro-2-propanol and solubilized with 70 μl of 70% formic acid, followed by sonication. Reversed-phase HPLC was performed on a C4-300 column (Tosoh, Tokyo, Japan) and an Äkta Basic P900 Protein Purifier. To elute the samples, a 0–70% acetonitrile/water gradient with 0.1% trifluoroacetic acid was used at room temperature for 30 min. Eluted samples were vacuum-dried, resolubilized in reducing sample buffer, and analyzed by SDS-PAGE and immunoblotting using α-Shh antibodies. Signals were quantified using ImageJ and normalized to the highest protein amount detected in each run.

### Shh Release Assays

Bosc23 cells were transfected with Shh or its C-terminally modified variants encoded in pcDNA3.1 using PolyFect and grown for 2 days. Afterward, cells were incubated in serum-free DMEM for 6 h followed by TCA precipitation of the supernatant. Supernatants and cell lysates were subjected to reducing SDS-PAGE and Western blot analysis using goat-α-Shh antibodies (R&D Systems), mouse α-HA (Sigma Aldrich) and rabbit α-StrepII antibodies followed by HRP conjugated secondary antibodies. Release was quantified by determining the ratio of soluble (detected in the supernatant) and cellular 19 or 46 kDa (Shh^*GFP*^) using ImageJ. The release of untagged Shh was set to 1. To determine the autocatalytic cholesteroylation efficiencies, ratios of processed 19 or 46 kDa protein vs. the unprocessed 46 or 75 kDa precursors of Shh, Shh^*HA*^, Shh^Δ*HA*^ Shh^*Strep*^, or Shh^*GFP*^ were determined using ImageJ. Ratios are expressed relative to untagged Shh, which was set to 1.

To study the effect of Scube2 on Shh release, Bosc23 cells were co-transfected with a bicistronic pIRES plasmid encoding Shh or its C-terminally modified variants and Hhat either with or without Scube2 or Scube2^Δ*CUB*^ cloned into pcDNA3.1 in a ratio 2:1. Cells were grown for 2 days and subsequently incubated in serum-free DMEM for 6 h. Supernatants were TCA precipitated, and Shh release was analyzed by reducing SDS-PAGE and immunoblotting. Shh release was quantified by determining the ratio of soluble Shh in the supernatant relative to Shh protein detected in the cell lysates. Quantification was done with ImageJ and Shh release without co-expression of Scube2 was set to 1.

### Bioactivity Assay

Bosc23 cells were grown and transfected with non-cholesterol modified ShhN or its C-terminal variants as described above. After 2 days, the supernatant was centrifuged to remove cell debris. C3H10 T1/2 reporter cells (Nakamura et al., 1997) were grown in DMEM containing 10% FCS and 100 μg/ml penicillin/streptomycin and seeded onto 24-well plates. Supernatants containing solubilized ShhN were immunoblotted to adjust protein concentration. Afterward, supernatants were diluted 1:1 with DMEM containing 10% FCS and antibiotics and applied to C3H10 T1/2 cells the following day. Cells were lysed 5 days after induction (PBS + 1% Triton X-100) and osteoblast-specific alkaline phosphatase activity was measured at 405 nm as previously described (Jakobs et al., 2016).

### Bioanalytical and Statistical Analysis

All statistical analyses were performed in GraphPad Prism. Applied statistical tests and post-tests are mentioned in the figure legends. A *P*-value of < 0.05 was considered statistically significant.

## Results

### Altered C-Terminal Peptides Interfere With Hh Short-Range Signaling Activity *in vivo*

**FIGURE 1:**
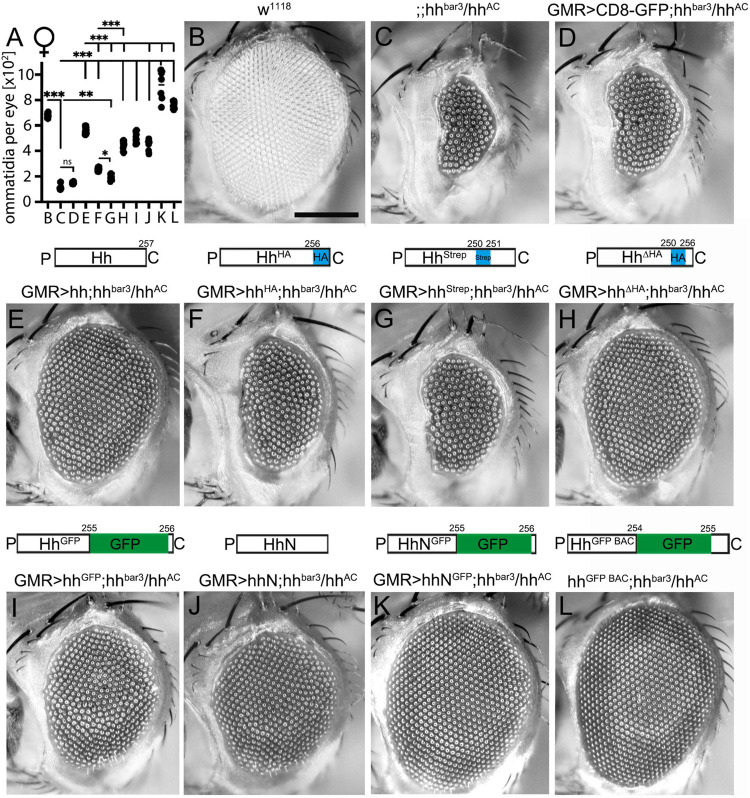
C-terminal peptide modifications variably modulate short-range signaling during Drosophila eye development at 18°C. **(A)** Quantification of ommatidia per eye of female flies shown in **(B–L)**. Statistical significance was determined by one-way ANOVA followed by Sidak’s multiple-comparison test. ***P < 0.001, **P < 0.01, *P < 0.05. ns: not significant. **(B)** Wild-type fly eyes (w^1118^) consist of several hundreds of ommatidia that develop in response to Hh signaling. Scale bar 100 μm. **(C)** In hh^*bar*3^/hh^*AC*^ female flies, eye development was strongly impaired. **(D)** As a negative control, CD8-GFP expressed in hh^*bar*3^/hh^*AC*^ flies under eye disc-specific GMR control did not rescue the hh^*bar*3^/hh^*AC*^ loss-of-function phenotype. **(E)** Expression of untagged Hh and **(F–I)** C-terminally modified Hh variants rescued eye development in hh^*bar*3^/hh^*AC*^ flies to varying degrees. **(J,K)** Non-cholesteroylated hhN and hhN^*GFP*^ partially rescued or fully restored eye development, respectively. **(L)** Hh^*GFP BAC*^ expression under endogenous promotor control also fully rescued eye development. All crossings were carried out at 18°C.

To determine cholesteroylated Hh biofunctions, we analyzed the following C-terminally tagged Hh variants: (i) Hemagglutinin tagged Hh (Hh^*HA*^, protein sequence YPYDVPDYA inserted between residues H256 and G257), (ii) a streptavidin tagged form (Hh^*Strep*^, protein sequence WSHPQFEK inserted between residues S250 and I251), and (iii) a variant having amino acids S250-H256 replaced with an HA tag (Hh^Δ*HA*^, Figure 1). *In vitro* expression in *Drosophila* S2 cells confirmed that all C-terminally modified Hh variants were produced and secreted to the cell surface (Supplementary Figure S1). We additionally analyzed two well-established C-terminally tagged Hh-GFP fusion proteins: (i) Hh^*GFP*^ generated and first published by I. Guerrero’s group (Torroja et al., 2004; Callejo et al., 2008, 2011; Bischoff et al., 2013) and (ii) Hh^*GFP BAC*^ obtained from T. Kornberg’s laboratory (Chen et al., 2017). Both Hh fusion constructs consist of the N-terminal, palmitoylated Hh protein linked to C-terminal GFP with a cholesterol moiety covalently attached to the GFP C-terminus (Figure 1).

All constructs were expressed in the *Drosophila* eye disc serving as one established *in vivo* model for Hh short-range signaling (Kastl et al., 2018). The adult *Drosophila* compound eye is comprised of several hundred facets (called ommatidia, Figures 1A,B) that develop in a wave of cell differentiation that moves across the eye disc epithelium from the posterior to the anterior side. The leading edge of this wave is marked by the morphogenetic furrow, a visible groove that demarcates the boundary between developing photoreceptors located posteriorly and undifferentiated cells located anteriorly. The movement of the morphogenetic furrow is guided by Hh secreted by posterior cells and sensed by anterior cells that in turn differentiate into posterior cells and also secrete Hh. This leads to furrow progression across the eye disc and determines the number of ommatidia in the adult eye (Ma et al., 1993). Accordingly, reduced Hh production in flies lacking most endogenous Hh production in the eye disc (in a hh^*bar*3^/hh^*AC*^ background) delays furrow progression and results in significantly fewer ommatidia (114 ± 19 in female flies at 18°C, Figures 1A,C) than in wild-type eyes (664 ± 35 ommatidia in female flies; *P* < 0.001, Figures 1A,B; Spratford and Kumar, 2014).

To rescue this loss-of-function phenotype, we used an established eye disc-specific glass multimer reporter (GMR)-Gal4 driver (Brand and Perrimon, 1993) or the endogenous promotor [for Hh^*BAC GFP*^ expression (Chen et al., 2017)] to drive expression of recombinant Hh or its C-terminally modified variants in hh^*bar*3^/hh^*AC*^ eye discs. As a negative control, CD8-GFP expression did not rescue the loss-of-function phenotype (152 ± 6 ommatidia in female flies; *P* > 0.05 compared with hh^*bar*3^/hh^*AC*^ flies, Figures 1A,D). In contrast, GMR-Gal4-controlled Hh expression almost restored eye development at 18°C (559 ± 25 ommatidia; Figures 1A,E). All GMR-Gal4-driven Hh variants significantly increased ommatidia number in hh^*bar*3^/hh^*AC*^ flies as well but failed to reach the level of unmodified Hh rescue (hh^*Strep*^: 195 ± 23 ommatidia; hh^*HA*^: 258 ± 12 ommatidia; hh^Δ*HA*^: 446 ± 34 ommatidia; *P* < 0.001 compared with hh; Figures 1A,F–H). The insertion of a large GFP tag (∼27 kDa) into the C-terminal peptide did not impair Hh short range bioactivity (hh^*GFP*^:506 ± 39 ommatidia; ns compared with hh; Figures 1A,I). Eye development was also restored upon hh^*GFP BAC*^ expression under minimal endogenous promotor control (hh^*GFP BAC*^: 765 ± 27 ommatidia at 18°C, Figures 1A,L).

Interestingly, the insertion of a small C-terminal Strep tag impaired short-range Hh bioactivity *in vivo* more strongly than did the insertion of an HA tag (hh^*Strep*^: 195 ± 23 ommatidia vs. hh^*HA*^: 258 ± 12 ommatidia, *P* < 0.05; Figure 1A). This indicates that the site of insertion or the amino acid sequence of the C-terminal tag affect Hh biofunction to different degrees. C-terminal peptide length is another determinant of Hh biofunction, because replacing the wild-type peptide downstream of S250 with an HA tag (hh^Δ*HA*^) increased bioactivity over that of hh^*HA*^ having the same tag inserted into the peptide (hh^Δ*HA*^: 446 ± 34 and hh^*HA*^: 258 ± 12 ommatidia in female flies, *P* < 0.001). Our results also confirm that the lipid moiety is not essential for Ptc receptor binding, because engineered monomeric, non-cholesterol modified HhN also rescued eye development (hhN: 451 ± 44 ommatidia, *P* < 0.001 compared with hh^*bar*3^/hh^*AC*^, Figures 1A,J), consistent with previous observations (Lewis et al., 2001; Taylor et al., 2001; Dawber et al., 2005). Moreover, Ptc receptor binding is apparently not affected by the presence of large C-terminal tags, as indicated by fully restored eye development as a consequence of non-cholesteroylated HhN^*GFP*^ expression (hhN^*GFP*^: 921 ± 116 ommatidia; Figures 1A,K). Rescue experiments in male flies matched those in female flies (hh: 416 ± 26 ommatidia; hh^Δ*HA*^: 347 ± 41 ommatidia; hh^*HA*^ 236 ± 30 ommatidia; hh^*Strep*^: 215 ± 25 ommatidia; hh^*GFP*^: 426 ± 30 ommatidia; hh^*GFP BAC*^: 677 ± 33 ommatidia; wild-type positive control: 594 ± 24 ommatidia; hhN: 383 ± 22 ommatidia; hhN^*GFP*^: 866 ± 86 ommatidia; hh^*bar*3^/hh^*AC*^ negative control: 154 ± 18 ommatidia; CD8-GFP expressing negative control: 180 ± 17 ommatidia, data not shown). When flies were kept at 25°C to increase GMR-Gal4-induced hh transgene expression (Supplementary Figure S2), Hh variant bioactivities approached those of untagged Hh, possibly due to saturation of the system. Supporting this possibility, Hh expression in hh^*bar*3^/hh^*AC*^ eye discs increased ommatidia numbers over those observed in wild-type flies [w^1118^: 632 ± 43 ommatidia vs. hh: 784 ± 25 ommatidia (in female flies, Supplementary Figure S2)]. All C-terminally modified Hh mutants rescued eye development in hh^*bar*3^/hh^*AC*^ eye discs to varying degrees, with Hh^*Strep*^ again being the least active (hh^*HA*^: 761 ± 19 ommatidia; hh^*Strep*^: 586 ± 41 ommatidia; hh^Δ*HA*^: 772 ± 16 ommatidia, hh^*GFP*^: 694 ± 39 ommatidia, hh^*GFP BAC*^: 819 ± 22 ommatidia, Supplementary Figure S2). Our findings thus show that the insertion of peptide or protein tags into the cholesteroylated C-terminal Hh peptide interferes with Hh regulated eye development to variable degrees, depending on the position and the sequence of the tag.

### Altered C-Terminal Peptides Variably Impair Hh Long-Range Signaling Activity *in vivo*

**FIGURE 2:**
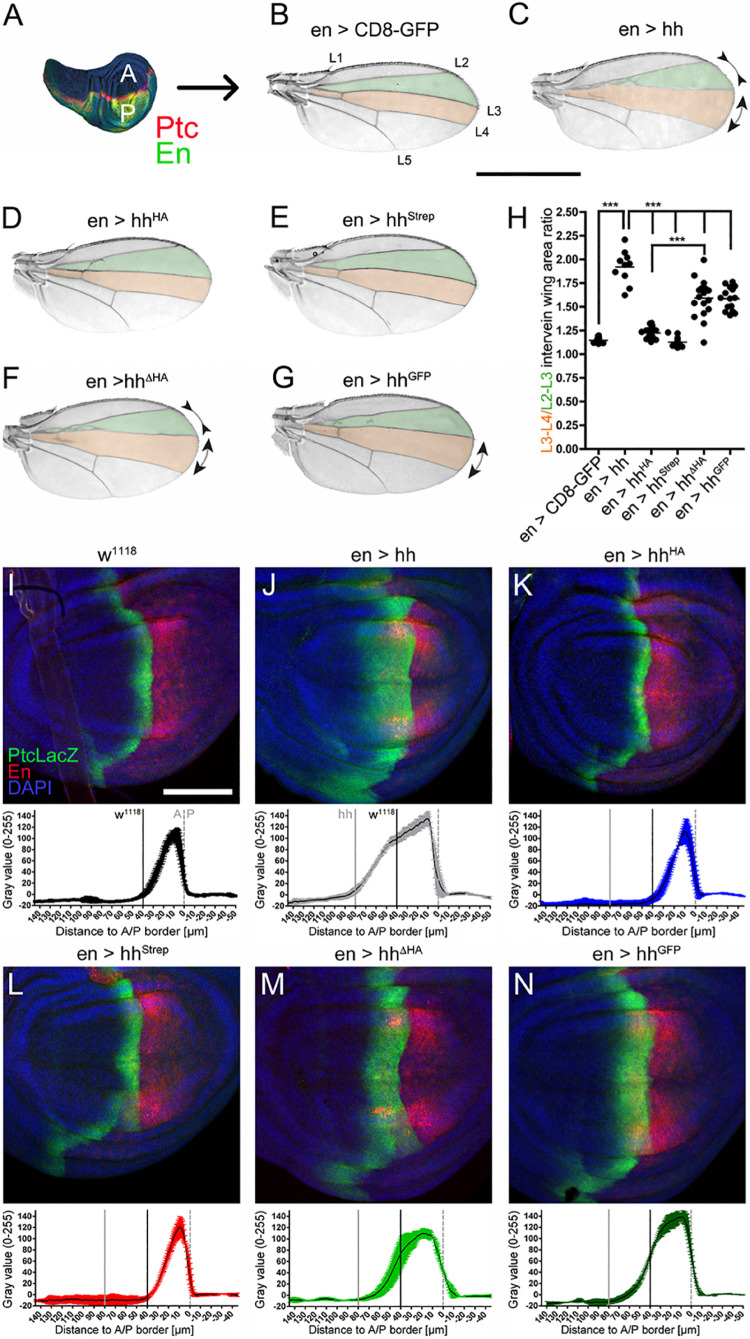
C-terminal modifications variably impair Hh long-range signaling in *Drosophila* wing development. **(A)** En-Gal4 drives Hh transgene expression in the posterior (P) compartment of the wing disc (green). Hh induces upregulation of Ptc in the anterior (A) compartment (red stripe). **(B)** Hh signaling directly patterns the L3–L4 intervein area (red) and indirectly the L2–L3 intervein area (green). Scale bar: 1 mm. **(C–G)** Adult wing patterning as a result of En-Gal4-controlled overexpression of hh or hh variants in the developing wing. **(H)** Quantification of **(B–G)** revealed significantly impaired Hh patterning activities of all C-terminally modified Hh variants compared to untagged Hh. Statistical significance was determined by one-way ANOVA followed by Bonferroni’s multiple-comparison test. ***P < 0.001. **(I–N)** Corresponding wing discs were stained against the anterior high- and medium- threshold target PtcLacZ (detected by anti-β-galactosidase staining, labeled green). The average distribution and intensity plots (*n* = 3 each; mean values ± SD) are also shown. Posterior compartments were intrinsically labeled using UAS-cherry (red). Scale bar: 100 μm.

Next, we investigated whether modified C-terminal peptides affect Hh long-range signaling activity in *Drosophila* wing development. In larval wing discs, Hh is expressed in the posterior compartment under the control of the transcription factor Engrailed (En) (Tabata et al., 1992; Zecca et al., 1995) and moves across the A/P boundary into the anterior compartment to bind to its receptor Ptc. High- and medium-threshold signlaing close to this boundary then induces downstream expression of the Hh target genes *en* and *ptc*, respectively (Crozatier et al., 2004). En is expressed in a stripe of five to seven anterior cells adjacent to the A/P border, and Ptc is upregulated in an ∼10 anterior cell stripe in response to medium Hh levels (Figure 2A; Strigini and Cohen, 1997). This directly patterns the central L3–L4 intervein region of the adult wing (Mullor et al., 1997; Strigini and Cohen, 1997; Figure 2B). We quantified wing phenotypes by dividing the L3–L4 areas by the L2–L3 areas. Consistent with previously published studies (Mullor et al., 1997; Lee et al., 2001; Kastl et al., 2018; Schürmann et al., 2018), En-controlled overexpression of recombinant Hh displaced the L3 vein anteriorly, resulting in an expanded L3–L4 intervein area, and concomitantly reduced the L2–L3 intervein area [en > CD8-GFP served as a negative control: L3–L4/L2–L3: 1.15 ± 0.03; en > hh: 1.92 ± 0.17 (+ 67%), *P* < 0.001; Figures 2C,H]. In contrast, En-Gal4-driven overexpression of C-terminally HA or Strep tagged Hh variants increased the L3–L4/L2–L3 ratio to a much lesser degree or not at all (en > hh^*HA*^: 1.22 ± 0.06 (−36% compared with en > hh) and en > hh^*Strep*^: 1.13 ± 0.04 (−41% compared with en > hh); Figures 2D,E,H). Similar to increased bioactivity in eye development, in transgenic flies overexpressing Hh^Δ*HA*^, the L3–L4/L2–L3 ratio was also enhanced compared to hh^*HA*^ (en > hh^Δ*HA*^: 1.6 ± 0.2, + 31% compared with en > hh^*HA*^, P > 0.001, but −17% compared with en > hh; Figures 2F,H). Similar L3–L4/L2–L3 ratio changes were observed in transgenic flies overexpressing hh^*GFP*^ under En-Gal4 control (1.57 ± 0.12, −18% compared with en > hh; Figures 2G,H). HhN expression from the same integration site and using the same driver line, and HhN^*GFP*^ expression were always lethal at 25 and 18°C, indicating ectopic or excessive signaling of both non-cholesteroylated transgenes.

Next, to confirm that observed wing mispatterning was specifically caused by altered Hh signaling, we analyzed Hh target gene expression at the molecular level. In wild-type flies (w^1118^), expression of the Hh target Ptc is upregulated in a narrow stripe of anterior cells located just adjacent to the A/P border (Figure 2I). Consistent with the observed expansion of L3–L4 areas in adult wings upon En-Gal4-controlled overexpression of Hh, we detected a broadened stripe of Ptc-expressing cells in larval wing discs (Figure 2J). Unaffected wing development, as observed in flies overexpressing hh^*HA*^ and hh^*Strep*^ under En-Gal4 control, corresponded with normal Ptc expression (Figures 2K,L), and moderate gain-of-function phenotypes observed in adult wings as a consequence of En-Gal4-controlled hh^Δ*HA*^ and hh^*GFP*^ overexpression corresponded with moderately upregulated Ptc expression in hh^Δ*HA*^ and hh^*GFP*^ wing discs (Figures 2M,N). Taken together, reduced wing disc patterning as a consequence of C-terminally tagged, dual-lipidated Hh variant expression indicates that altered cholesteroylated peptide sequence or length modifies Hh biofunction also over the long range, similar to what has been observed for differently modified N-terminal peptides in the same system (Kastl et al., 2018).

### C-Terminal Insertional Mutagenesis Attenuates Dominant-Negative ^*C*85S^Hh Activity *in vivo*

**FIGURE 3:**
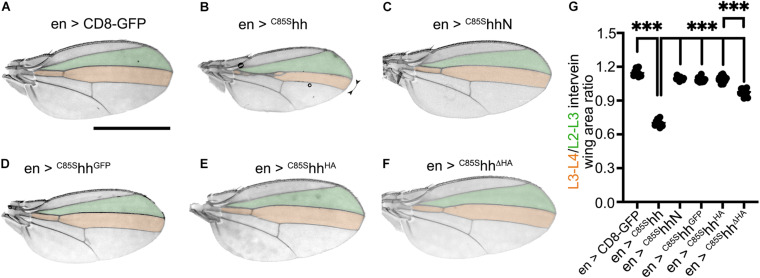
C-terminal tags attenuate dominant-negative ^*C*85S^Hh function *in vivo*. **(A–F)** Wing patterning as a consequence of En-Gal4-controlled overexpression of non-palmitoylated Hh variants. Scale bar: 1 mm. **(G)** Quantification of **(A–F)**. Statistical significance was determined by one-way ANOVA followed by Sidak’s multiple comparison test. ***P < 0.001.

We based a third set of *in vivo* experiments on the known, yet poorly understood dominant-negative activity of cholesteroylated yet non-palmitoylated ^*C*85S^Hh variants on endogenous Hh, if co-expressed in the same (posterior) wing disc compartment (Lee et al., 2001). As a consequence of dominant-negative ^*C*85S^Hh activity, the size of the proximal L3–L4 intervein area of adult wings was reduced [en > CD8-GFP: L3–L4/L2–L3 = 1.15 ± 0.03; en > ^*C*85S^hh: L3–L4/L2–L3 = 0.7 ± 0.03 (–40% compared with en > CD8-GFP, *P* < 0.001) Figures 3A,B,G]. The additional deletion of C-cholesterol (resulting in non-lipidated ^*C*85S^HhN) resulted in soluble morphogens that did not impair endogenous Hh function (Schürmann et al., 2018; Figures 3C,G). This suggests that cholesterol is required for dominant-negative ^*C*85S^Hh suppression of endogenous Hh function if both proteins are expressed in the same compartment. However, we observed only mildly affected wing patterning in flies expressing GFP tagged ^*C*85S^Hh^*GFP*^, despite its cholesteroylation [en > ^*C*85S^hh^*GFP*^: L3–L4/L2–L3 = 1.09 ± 0.02 (+ 50% compared with en > ^*C*85S^hh, *P* < 0.001) Figures 3D,G]. Similar attenuation of Hh activity suppression was observed when a small HA tag was C-terminally inserted into the non-palmitoylated, yet cholesteroylated protein [en > ^*C*85S^hh^*HA*^: L3–L4/L2–L3 = 1.10 ± 0.02 (+ 55% compared with en > ^*C*85S^hh, *P* < 0.001) Figures 3E,G]. Replacing the C-terminal peptide with an HA tag (in ^*C*85S^Hh^Δ*HA*^), however, increased the dominant-negative activity of the non-palmitoylated protein [en > ^*C*85S^hh^Δ*HA*^: L3–L4/L2–L3 = 0.98 ± 0.02 (+ 39% compared with en > ^*C*85S^hh, *P* < 0.001), Figures 3F,G]. Thus, similar to what we have observed for eye and wing development, replacing the C-terminal peptide with an HA tag affected protein biofunction less than insertion of the same tag to extend the Hh C-terminus [en > ^*C*85S^hh^*HA*^: L3–L4/L2–L3 = 1.10 ± 0.02 vs. en > ^*C*85S^hh^Δ*HA*^: L3–L4/L2–L3 = 0.98 ± 0.02 (−11% compared with en > ^*C*85S^hh^*HA*^, *P* > 0.001), Figures 3E–G].

These observed differences were even more pronounced when Hh-Gal4 instead of En-Gal4 was used to alternatively drive transgene expression in the same posterior wing disc compartment (Supplementary Figure S3): L3–L4/L2–L3 intervein areas of hh > CD8-GFP flies were essentially of wild-type size (1.2 ± 0.04), yet those of hh > ^*C*85S^hh flies were reduced to 0.47 ± 0.04 (–60% compared with hh > CD8-GFP, *P* < 0.001). Again, the intervein areas of hh > ^*C*85S^hh^*GFP*^ and hh > ^*C*85S^hh^*HA*^ flies were much less reduced than those of hh > ^*C*85S^hh flies and approached those of hh > CD8-GFP flies (^*C*85S^hh^*GFP*^: 1.16 ± 0.04, + 145% compared with hh > ^*C*85S^hh; ^*C*85S^hh^*HA*^: 1.12 ± 0.02, + 137% compared with hh > ^*C*85S^hh; hh > CD8-GFP: + 155% compared with hh > ^*C*85S^hh, Supplementary Figure S3F). Again, replacing the C-terminal sequence with an HA tag reduced the dominant-negative activity less (^*C*85S^hh^Δ*HA*^: 0.98 ± 0.03, + 108% compared with hh > ^*C*85S^hh, Supplementary Figures S3E,F, and –14% compared with hh > ^*C*85S^hh^*HA*^, *P* < 0.001). These findings suggest that (i) dominant-negative wing patterning phenotypes as a consequence of ^*C*85S^Hh expression in the posterior wing disc compartment are not a direct consequence of the lack of N-palmitate and (ii) that although cholesterol is required for dominant-negative ^*C*85S^Hh suppression of endogenous Hh function, this function also depends on the unchanged length and sequence of the associated C-terminal Hh peptide.

### C-Terminal Insertional Mutagenesis Reduces Dominant-Negative Activities of N-Terminally Tagged Hh *in vivo*

**FIGURE 4:**
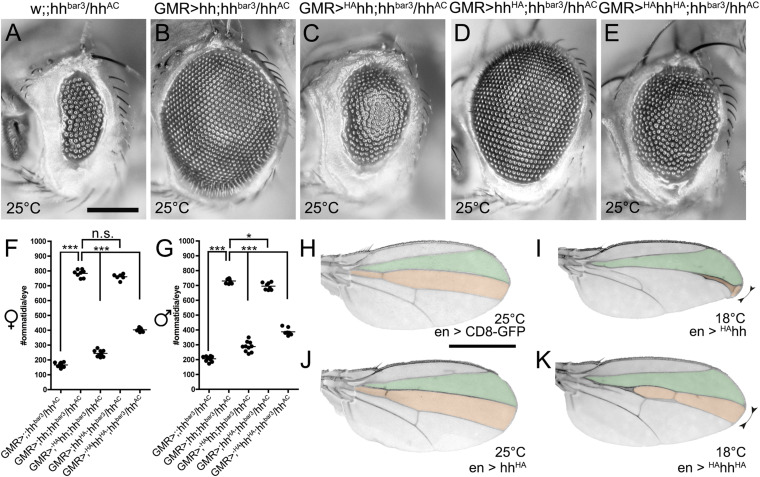
N- and C-terminal insertion of HA tags affect Hh biofunction to different degrees. **(A–E)** Eye phenotypes in **(A)** hh^*bar*3^/hh^*AC*^ flies that express **(B)** untagged hh, **(C)** N-terminally HA tagged ^*HA*^hh, **(D)** C-terminally HA tagged hh^*HA*^, or **(E)** N- and C-terminally tagged ^*HA*^hh^*HA*^, all under GMR-control at 25°C. Scale bar: 100 μm. **(F,G)** Quantification of female or male fly eyes shown in **(A–E)**. Statistical significance was determined by one-way ANOVA followed by Tukey’s multiple-comparison test. ***P < 0.001, *P < 0.05, *P* > 0.05 was considered as not significant (n.s.) **(H–K)** Representative wing patterning in flies expressing **(H)** CD8-GFP as a negative (inactive) transgene control, **(I)** N-terminally HA tagged ^*HA*^hh that suppresses the activity of endogenous Hh expressed in the same compartment, **(J)** C-terminally HA tagged hh^*HA*^, or **(K)** dually HA tagged ^*HA*^hh^*HA*^, all under En-Gal4 control. Because N-terminal HA tagging resulted in pharate lethality at 25°C, the experiment was conducted at 18°C. Scale bar: 1 mm.

Next, we compared possible interference of N- or C-terminal peptide tagging *in vivo*, again using eye development as a read-out for short-range Hh biofunction. We observed that, unlike the untagged protein, N-terminally HA tagged ^*HA*^Hh expressed under GMR-Gal4-control improved the hh^*bar*3^/hh^*AC*^ loss-of-function phenotype in eye discs only mildly (hh^*bar*3^/hh^*Ac*^: 166 ± 15 ommatidia in female flies; hh: 784 ± 25 ommatidia; ^*HA*^hh: 244 ± 22 ommatidia; Figures 4A–C,F,G), even if strongly expressed at 25°C (Kastl et al., 2018). In contrast, the same HA tag inserted into the cholesteroylated C-terminal peptide affected short-range signaling at 25°C much less (hh^*HA*^: 761 ± 20 ommatidia; Figures 4D,F,G). Notably, a variant having both terminal peptides tagged (^*HA*^hh^*HA*^) partially restored Hh loss of function caused by the N-terminal tag (^*HA*^hh^*HA*^: 403 ± 13 ommatidia; Figures 4E–G). Similar biological effects of HA tagged Hh variant expression were also observed for Hh long-range signaling during wing development: En-Gal4-controlled expression of N-terminally modified ^*HA*^hh caused severe loss-of-function wing phenotypes, while hh^*HA*^ expression affected wing development only mildly or not at all (Figure 4J), and expression of ^*HA*^hh^*HA*^ resulted in an intermediate dominant-negative wing phenotype (Figure 4K).

### Inserted C-Terminal Peptide Tags Affect Ligand Multimerization and Are Prone to Proteolytic Cleavage *in vivo* and *in vitro*

**FIGURE 5:**
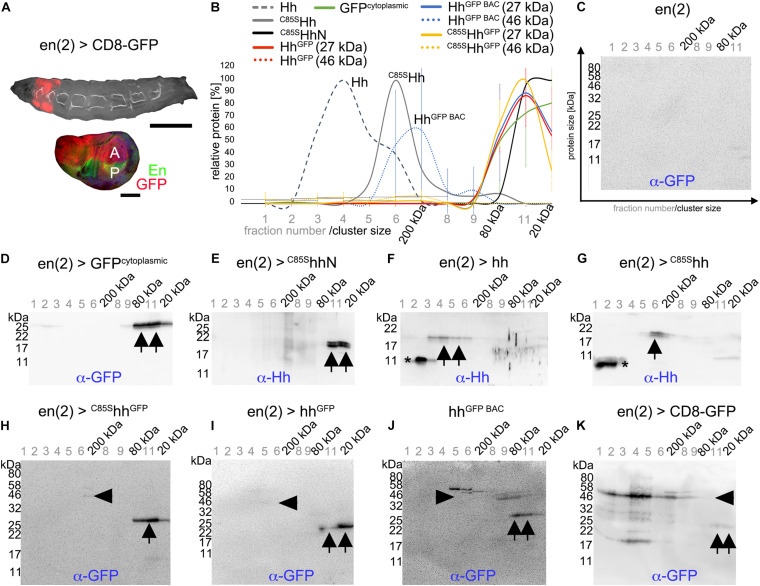
Hh C-terminal GFP tags are proteolytically processed and monomeric *in vivo.* **(A)** En(2)-Gal4 drives transgene expression in both anterior and posterior compartments in larval imaginal discs (red). Scale bars: 500 and 100 μm, respectively. **(B)** SEC of larval homogenates expressing lipidated or unlipidated Hh or GFP tagged Hh variants under En(2)-Gal4 control, followed by immunoblotting with α-GFP or α-Hh antibodies. *n* = 3 for all C-terminally modified Hh variants. Mean values ± SD are shown. **(C–K)** Representative examples of SEC analyses used for **(B)**. Asterisks indicate unspecific staining, arrowheads denote Hh-GFP or CD8-GFP fusion proteins, and arrows indicate specific α-GFP or α-Hh staining of processed proteins.

How can Hh activity modulation by ^*C*85S^Hh and ^*HA*^Hh and its attenuation by inserted C-terminal tags be explained? It is known that prior to their release, Hhs form large clusters at the surface of producing cells (Vyas et al., 2008). One possibility therefore is that ^*C*85S^Hh and ^*HA*^Hh integrate into these clusters to suppress endogenous Hh release (Kastl et al., 2018) or bioactivation (Schürmann et al., 2018) and that additional C-terminal modifications interfere with this process. To test this idea, we first analyzed the capacity of tagged Hh variants to multimerize *in vivo*. To this end, we used size exclusion chromatography (SEC) fractionation of transgenic En(2)-Gal4 driven expression [en(2) >] of Hh and Hh^*GFP*^ variants in *Drosophila* larvae (Figure 5A) followed by reducing SDS-PAGE and immunoblotting using α-GFP or α-Hh antibodies (Figures 5B–K). Homogenates of larvae expressing Gal4 alone served as negative controls (Figure 5C). Positive control en(2) > GFP^*cytoplasmic*^ from L3 larval homogenates eluted in the last three SEC fractions, indicating the presence of monomeric (27 kDa) GFP as well as small GFP clusters (dimers or trimers) (Figures 5D arrows, B). A similar size distribution was observed for non-lipidated monomeric ^*C*85S^HhN (Figures 5E arrows, B). In contrast, authentic dual-lipidated multimeric Hh and non-palmitoylated yet cholesterol-modified ^*C*85S^Hh were mainly found in fractions 3–6 (Figures 5F,G,B), corresponding to MWs of 100–200 kDa as previously reported (Gallet et al., 2003, 2006). In contrast to Hh and ^*C*85S^Hh, most α-GFP reactive proteins obtained from en(2) > ^*C*85S^hh^*GFP*^ larvae eluted in fractions 10–12, suggesting MWs of the non-denatured proteins of only ∼27–80 kDa (Figures 5H arrows, B). Notably, SDS-PAGE and immunoblotting of (denatured) proteins detected mainly ∼27 kDa GFP (compare Figures 5D,H) and only very small amounts of multimeric intact ∼46 kDa ^*C*85S^Hh^*GFP*^ (Figure 5H arrowhead). Therefore, in addition to impaired ^*C*85S^Hh^*GFP*^ multimerization, this finding indicates extensive proteolytic processing of the C-terminal peptide that links Hh with the 27 kDa GFP tag. We confirmed this finding for GFP tagged Hh variants Hh^*GFP*^ (Torroja et al., 2004) and Hh^*GFP BAC*^ (Chen et al., 2017): Here, too, we mostly detected processed 27 kDa GFP without its N-terminal Hh (Figures 5I,J arrows, B). To rule out that GFP tags generally undergo extensive unspecific processing under the experimental conditions used in our work, we analyzed CD8-GFP-expressing larvae as controls. In contrast to all GFP-linked Hh variants, CD8-GFP remained multimeric and largely intact (∼50 kDa; Figure 5K arrowhead), and only little separated GFP was found (Figure 5K, arrows). These experiments suggested that Hh C-termini are prone to *in vivo* cleavage, which would separate Hh morphogens from their cholesterol moieties. We also attempted to directly confirm this possibility *in vivo* by double-staining Hh^*GFP*^ in wing discs with α-Hh and α-GFP antibodies, expecting the presence of both signals in the posterior wing disc compartment, yet only the Hh signal—and no GFP—in the anterior receiving compartment. Although anterior GFP was not detected, as expected, insufficient α-Hh antibody sensitivity prevented microscopic Hh signal detection in both compartments (data not shown).

We therefore used another α-GFP antibody to validate proteolytic Hh^*GFP*^ processing in larval homogenates (Supplementary Figures S4A–C). Although this antibody was more sensitive, only little Hh-GFP fusion proteins (Supplementary Figure S4C arrowhead) relative to much higher amounts of monomeric cleaved GFP were detected in the fractions (Supplementary Figure S4C arrow). We also analyzed isolated larval disc tissue that expressed the transgenic proteins (instead of whole larval lysates) and obtained the same results (Supplementary Figure S4D). Additionally, we distinguished between soluble and insoluble proteins in larval homogenates by using detergent-based or detergent-free lysis buffers. More intact Hh-GFP fusion proteins were found in the presence of Triton-X100 (Supplementary Figures S4E,F, arrowheads), indicating their cell membrane association, while separated 27 kDa GFP was detected under both conditions (Supplementary Figures S4E,F arrows). These findings support that the peptide connecting Hh and GFP is prone to proteolytic cleavage *in vivo*. Finally, to ensure that SEC conditions did not cause the observed protein cleavage, we directly lysed transgenic larvae in hot reducing SDS buffer and immediately analyzed the crude lysates by SDS-PAGE/Western blot. Again, we detected substantial amounts of processed GFP and little amount of intact Hh-GFP fusion proteins (Supplementary Figures S4G,H). Yet, we note that high levels of processed GFP, if compared with the membrane-associated full-length fusion protein, might have resulted from the accumulation of stable GFP during L1–L3 larval development and therefore may not represent the actual extent of ^*C*85S^Hh^*GFP*^ proteolysis in L3 larvae. This is supported by our inability to detect (cleaved) 19 kDa Hh in the same samples, indicating that Hh is more rapidly removed from the system than the GFP tag. Still, our findings indicate extensive proteolytic C-terminal Hh cleavage as well as impaired Hh^*GFP*^ clustering *in vivo.*

**FIGURE 6:**
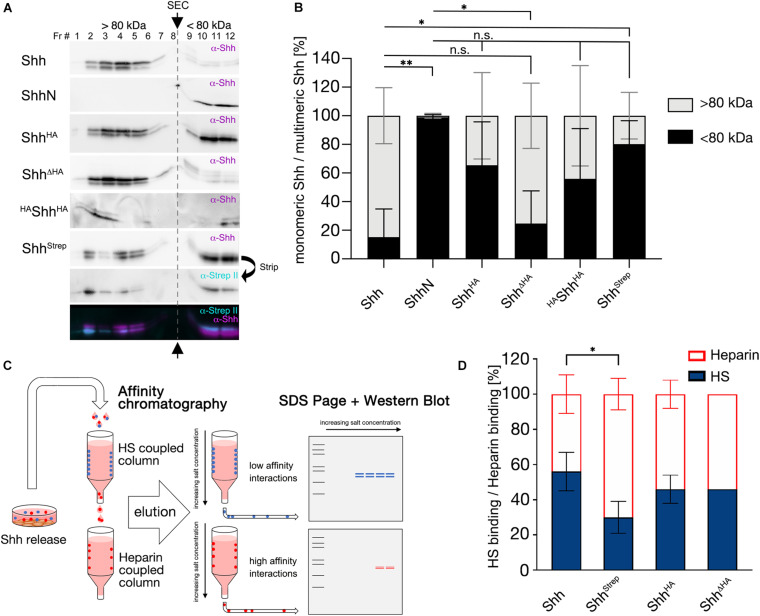
C-terminal tags impair Hh multimerization and HS binding *in vitro.* **(A)** SEC of soluble Shh secreted from Bosc23 cells. Fractions 1–8 (> 80 kDa) denote multimeric Shh and fractions 9–12 (< 80 kDa) monomeric Shh. Arrows and the gray dashed line separate monomeric and multimeric Shh. Insertional mutagenesis (Shh^*HA*^, ^*HA*^Shh^*HA*^, and Shh^*Strep*^) variably shifted multimerization toward monomeric Shh (compare with monomeric ShhN) compared with untagged Shh. Replacing the C-terminal Hh peptide restored multimerization *in vitro*. **(B)** Quantification of **(A)**. Black bars represent monomeric Shh protein and light gray bars multimeric Shh. Mean values ± SD are shown; *n* ≥ 3. Statistical significance was determined by one-way ANOVA followed by Sidak’s multiple-comparison test. n.s not significant, *P < 0.05, **P < 0.01. **(C)** Schematic of tandem affinity chromatography. Supernatant was first subjected to a low-sulfated HS-coupled column to immobilize strongly interacting proteins (blue). Unbound proteins in the flowthrough (low-affinity HS binders, red) were directly applied to a coupled heparin column. Afterward, both columns were separated, the bound material eluted with increasing salt concentrations and subsequently immunoblotted. **(D)** Quantification of **(C)**. Insertion of a C-terminal Strep tag significantly impaired protein binding to (low-sulfated) HS relative to non-physiological (high-sulfated) heparin. Mean values ± SD are shown; *n* > 3. Statistical significance was determined by one-way ANOVA followed by Dunnett’s multiple-comparison test. *P < 0.05.

Although we also intensively aimed to analyze *in vivo* multimerization of HA and Strep tagged Hh variants, α-Hh antibodies, α-HA antibodies, and α-StrepII antibodies failed to reliably detect Hh variants in larval lysates and cross-reacted strongly with *Drosophila* proteins (not shown). Therefore, we expressed HA and Strep tagged vertebrate Shh variant proteins in Bosc23 cells—a HEK 293T derivate—and subjected serum-containing supernatants to SEC. Using this method, we detected Shh mainly in fractions 2–5 (corresponding to MWs exceeding 200 kDa, Figure 6A upper row,B, Shh: 15 ± 20% < 80 kDa), in good agreement with the extent of Hh multimerization *in vivo* (Figures 5F,B), and detected soluble non-cholesteroylated ShhN in fractions 10–12, as expected for monomeric proteins or protein dimers/trimers (ShhN: 99 ± 2% < 80 kDa, *P* < 0.01 compared with Shh, Figure 6A second row,B). In contrast to wild-type Shh, α-Shh antibodies detected C-terminally tagged Shh variants Shh^*Strep*^, Shh^*HA*^, and ^*HA*^Shh^*HA*^ (the latter representing ^*HA*^Hh^*HA*^ as shown in Figure 4) in multimeric (> 80 kDa) as well as in monomeric (< 80 kDa) forms (Shh^*HA*^: 65 ± 30% < 80 kDa, Shh^*Strep*^: 80 ± 16% < 80 kDa, *P* < 0.05 compared with Shh, ^*HA*^Shh^*HA*^: 56 ± 35% < 80 kDa, Figures 6A,B). Shh^Δ*HA*^ multimerization was not changed (Shh^Δ*HA*^: 25 ± 23% < 80 kDa, Figures 6A,B). One possible explanation for these findings is that small inserted C-terminal tags may impair but do not fully abolish Shh multimerization, while multimerization is not affected when the C-terminal Hh peptide is replaced by the tag. However, we also observed that most monomeric Shh^*HA*^, ^*HA*^Shh^*HA*^, and Shh^*Strep*^ were terminally cleaved, similar to what we have observed for GFP tagged Hh variants *in vivo* that had their hydrophobic cholesterol moieties removed together with the tag (Figure 6A merged figure and Supplementary Figure S6). A second possibility to explain the generation of soluble monomeric proteins therefore is by the facilitated proteolytic removal of lipidated terminal peptides as a consequence of C-terminal tagging, which would in turn prevent Hh/Shh multimerization. Both possible explanations—impaired multimerization due to steric constraints imposed by the tag or facilitated proteolytic ligand processing and loss of cholesterol required for multimerization—are in line with our observed attenuation of dominant negative eye and wing phenotypes as a consequence of C-terminal Hh tagging, because both would limit transgene interactions with endogenous Hh at the surface of producing cells.

### C-Terminal Insertional Mutagenesis Can Reduce HS Binding

Another known *in vivo* contributor to Hh biofunction is heparan sulfate (HS) (The et al., 1999; Han et al., 2004; Vyas et al., 2008; Ortmann et al., 2015). HS is ubiquitously expressed at the cell surface and in the extracellular matrix (Sarrazin et al., 2011) and consists of a linear arrangement of negatively charged sugar residues that interact with positively charged Hh amino acids (Rubin et al., 2002). We therefore asked whether the insertion of C-terminal tags affects Hh binding to HS. To answer this question, we expressed Shh or tagged Shh variants in Bosc23 cells and subjected the supernatant to tandem affinity chromatography (Figure 6C). The first column contained low-sulfated HS isolated from *Drosophila melanogaster* as the immobile matrix (Figure 6C; Kusche-Gullberg et al., 2012). The Shh flowthrough from this column was directly applied to a connected heparin column. Heparin is a highly negatively charged form of HS that strongly binds and immobilizes proteins with lower positive charge (Figure 6C; Zhang et al., 2007). Immobilized protein variants were eluted separately and subjected to SDS-PAGE and Western blotting. We then calculated the amount of HS-eluted Shh relative to the total Shh amount eluted from both columns. Approximately half of untagged Shh bound to HS (56 ± 11%, Figure 6D) and half bound to heparin, whereas only one third of Shh^*Strep*^ was immobilized by low-sulfated HS (30 ± 9%, −46% compared with Shh, *P* < 0.05, Figure 6D). However, inserting an HA tag or replacing the C-terminal stem by an HA tag did not significantly affect HS binding (Shh^*HA*^: 46 ± 8%; −18% compared with Shh; Shh^Δ*HA*^: 46 ± 0%, −18% compared with Shh). This indicates that depending on the position and/or the sequence of the C-terminal tag, HS binding might or might not be reduced if compared to wild-type Shh. These reductions, however, do not correlate with reduced bioactivities of our mutants in gain-of-function experiments (Figure 2), nor their changed dominant-negative activities (Figure 3).

### C-Terminal Peptide Mutagenesis Affects Shh Precursor Autocatalytic Cholesteroylation and Regulated Shh Release but Does Not Interfere With Ptc Receptor Binding

**FIGURE 7:**
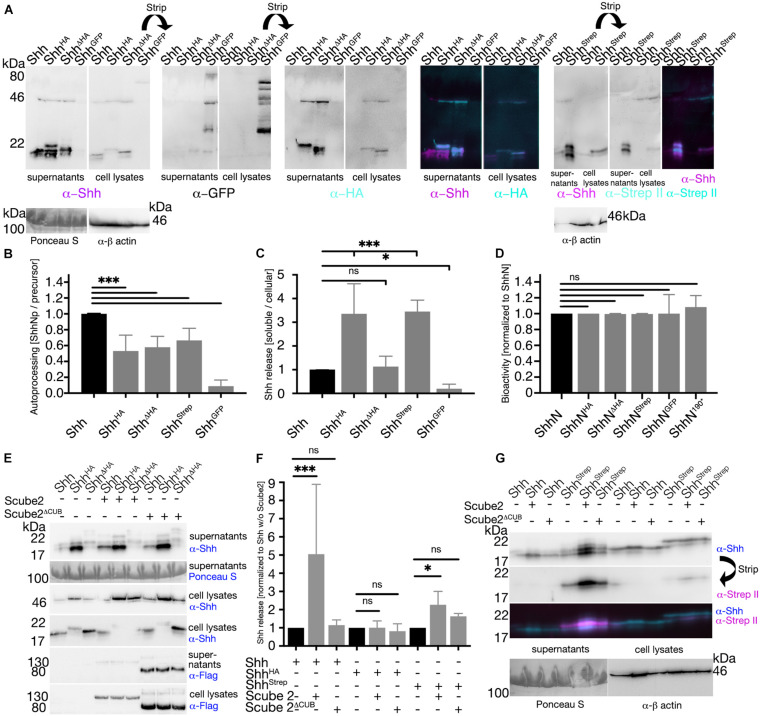
C-terminal tags impair ShhNC autocatalytic cholesteroylation and regulated Shh release. **(A)** Representative Western blots of C-terminally modified Shh variants. Solubilized Shh (supernatant) and cellular Shh (cell lysates) were blotted and stained with antibodies directed against Shh or its C-terminal tags. **(B)** Quantification of autocatalytic cholesteroylation efficiencies. Ratios of processed cellular Shh relative to precursors were determined and processing of the untagged protein was set to 1 (black bar). **(C)** Quantification of Shh release. Ratios of soluble (supernatant) Shh relative to cellular (cell lysate) Shh were determined and untagged protein release was set to 1 (black bar). Mean values ± SD are shown. Statistical significance was determined by one-way ANOVA followed by Dunnett’s multiple-comparison test. ns: not significant *P < 0.05 ***P < 0.001. **(D)** Bioactivity of Shh and its C-terminally modified variants. Non-cholesterol modified but C-terminally tagged Shh was expressed in Bosc23 cells and subsequently induced osteoblast differentiation of C3H10 T1/2 reporter cells. C-terminal protein tags did not interfere with Ptc receptor binding. **(E,G)** Representative Western blots of Shh or its variants with or without Scube2 and inactive Scube2^Δ*CUB*^. **(F)** Quantification of **(E,G)**. Mean values ± SD are shown in all graphs. Statistical significance was determined by one-way ANOVA followed by Dunnett’s multiple-comparison test. ns: not significant, *P < 0.05, ***P < 0.001.

We also analyzed whether small C-terminal tags interfere with the first post-translational Hh modification, the autocatalytic cholesteroylation of 45 kDa ShhNC precursor proteins in the ER. To this end, Shh variants were expressed in Bosc23 cells and the ratios of autocatalyzed proteins relative to their precursors were determined (Figure 7A). We found significantly impaired autocatalytic cholesteroylation of all C-terminally tagged Shh variants if compared with untagged Shh (Shh^*HA*^: 0.53 ± 0.2; Shh^Δ*HA*^: 0.58 ± 0.1; Shh^*Strep*^: 0.67 ± 0.15; Shh^*GFP*^: 0.09 ± 0.08, P < 0.001, Figure 7B). Notably, if processed, all 20 kDa Shh variants were C-terminally cholesteroylated, consistent with strictly coupled reactions of Hh autoprocessing and cholesterolylation of the Hh signaling domain (Porter et al., 1996a,b), and as confirmed by similar hydrophobicities of 20 kDa ^*C*25A^Shh^*HA*^ and 19 kDa untagged ^*C*25S^Shh (Supplementary Figure S5A) as well as increased electrophoretic mobility of the cholesteroylated proteins (Supplementary Figure S5B). The insertion of GFP into the C-terminal Shh peptide affected autocatalytic cholesteroylation most strongly (Figures 7A,B) as has been reported before (Chamberlain et al., 2008). Next, we analyzed the effect of C-terminal peptide modifications on Shh “background” (e.g., unregulated) release from the cell surface into the medium. The insertion of an HA tag or a Strep tag (in Shh^*HA*^ or Shh^*Strep*^) strongly increased background protein release, in contrast to the replacement of C-terminal amino acids with the HA tag (Shh^Δ*HA*^) that did not enhance unregulated release (Figures 7A,C). This suggests that, in the absence of known activators of Hh solubilization (such as Scube2), C-terminally tagged Shh variants are prone to C-terminal processing at the cell surface, consistent with what we have found *in vivo* (Figure 5) and *in vitro* (Figure 6). Of note, we observed differently sized soluble Shh, Shh^*HA*^, Shh^Δ*HA*^, and Shh^*Strep*^ originating from one cellular precursor, and only the highest MW soluble protein retained the tag (Figure 7A, merged image). This indicates that proteolytic Shh processing can occur upstream or downstream of the tags. In contrast to the insertion of small tags, inserting a C-terminal GFP tag not only drastically impaired autocatalytic ShhNC precursor processing but also reduced proteolytic Shh^*GFP*^ release from producing cells (Figures 7A,C). Finally, we found that the overall length of the non-cholesteroylated C-terminal peptide did not directly affect Shh bioactivity: All soluble, non-cholesteroylated but C-terminally tagged ShhN forms induced osteoblast differentiation in reporter cells to similar degrees *in vitro* (Figure 7D), which is in good agreement with unimpaired HhN^*GFP*^ short-range activity *in vivo* (Figure 1K). This rules out impaired Hh folding and any inhibited interactions of solubilized ligand with its receptor Ptc as possible causes for the reduced bioactivity of tagged variants *in vivo*.

Finally, we analyzed Scube2-regulated Shh and Shh variant release from the outer cell membrane of producing cells. Consistent with the described protease-enhancing activity of CUB domains (Gaboriaud et al., 2011), Scube2 increased proteolytic Shh processing from the cell surface into the medium over background levels, whereas co-expression of Scube2^Δ*CUB*^ engineered to lack the CUB domain did not enhance proteolytic Shh processing (Shh + Scube2: 5.1 ± 3.8; Shh + Scube2^Δ*CUB*^: 1.1 ± 0.3 compared with Shh in the absence of Scube2; Figures 7E,F). As observed before (Figures 7A,C), Shh^*HA*^ and Shh^*Strep*^ background release was higher than that of untagged Shh (Figures 7E,G) and release of both tagged forms was less affected by co-expression of Scube2 than release of untagged Shh (Shh^*HA*^ + Scube2: 1.1 ± 0.4; Shh^*HA*^ + Scube2^Δ*CUB*^: 0.8 ± 0.4 compared with Shh^*HA*^ in the absence of Scube2; Shh^*Strep*^ + Scube2: 2.3 ± 0.7; Shh^*Strep*^ + Scube2^Δ*CUB*^: 1.5 ± 1.6 compared with Shh^*Strep*^ in the absence of Scube2; Figures 7E–G). This indicates that C-terminal insertional mutagenesis can render Shh insensitive to Scube2 function, potentially by disturbing the regulated release of tagged Shh variants from the surface of producing cells. Dysregulated proteolytic C-terminal Hh processing, resulting in increased unregulated background release and reduced regulated release, may thus have contributed to our observed *in vivo* mispatterning phenotypes.

## Discussion

Directly or indirectly, the unique cholesterol modification serves several functional roles in Hh biology. Its first decisive role is to tether the nascent protein to membranes to prevent its unregulated release (Porter et al., 1996a; Lewis et al., 2001; Gallet et al., 2006; Gallet, 2011) and to increase interactions with the transmembrane-palmitoyltransferase Hhat for efficient Hh N-palmitoylation (Konitsiotis et al., 2015). Other suggested roles are to facilitate HS-assisted Hh multimerization (Vyas et al., 2008; Jakobs et al., 2016) and to tether Hh to lipophorin, Scube2, exosomes, or cytonemes (Torroja et al., 2004; Panakova et al., 2005; Tukachinsky et al., 2012; Gradilla et al., 2014; Parchure et al., 2015; Chen et al., 2017) for subsequent Ptc receptor binding and signaling (Qian et al., 2019). Although cholesterol may contribute directly to all of these processes, the interdependency of Hh production, multimerization, release, and signaling also raises the alternative possibility that cholesterol is directly required for initial steps of morphogen/membrane association and multimerization but contributes only indirectly to later steps of Hh signaling. The following findings support this alternative possibility.

### C-Terminal Peptide Structure and Sequence Variably Affect Hh Function *in vivo*

Our initial finding was that C-terminal tags reduced Hh signaling to different degrees. One underlying reason likely is that C-terminal peptide modifications affect autocatalytic cholesteroylation during protein biosynthesis, consistent with a previous report (Chamberlain et al., 2008). However, similar maturation yet different bioactivities of Hh variants with small peptide tags (Figure 7B) indicate that other factors, such as impaired multimerization or multimer half-life, may also contribute to the observed activity variations. Notably, we found that large GFP tags impaired Hh short range and long-range activity only mildly or not at all. Indeed, Hh^*GFP BAC*^ has previously been described to restore the full developmental program in hh^*AC*^ amorphs, leading to viable flies with normal morphology (Chen et al., 2017), and our study confirms near-physiological Hh^*GFP BAC*^ activity during eye development in a strong Hh loss-of-function background. We point out that such similar Hh and Hh^*GFP*^ ^*BAC*^ biofunctions *in vivo* are difficult to reconcile with suggested modes of Disp-mediated Hh membrane-extraction, hand over from Disp to suggested soluble carriers such as Scube2 in vertebrates (Tukachinsky et al., 2012), and subsequent relay and receptor activation during development (Qian et al., 2019). One perceived difficulty with this model is that the 27 kDa GFP tag imposes sterical restrictions on the associated 19 kDa Hh protein that likely interfere with direct Hh binding to their suggested partner molecules. A second perceived difficulty is that, in Hh^*GFP BAC*^, the cholesterol moiety is attached to the C-terminal of GFP. This is expected to severely affect suggested modes Disp-mediated Hh cholesterol extraction from the membrane and its subsequent binding to the Scube2 CUB-domain as suggested in vertebrates (Tukachinsky et al., 2012) and the receptor Ptc (Qian et al., 2019). Unimpaired Hh^*GFP BAC*^ activity in this model would therefore requires that Disp, hypothesized Scube2-like Hh carriers and Ptc can fully adapt to the increased protein size, changed multimerization, and lipid transfer to the tag, a scenario that we consider as not very likely.

### Tagged and Untagged Hhs Undergo C-Terminal Hh Processing

An alternative explanation for largely unimpaired Hh^*GFP BAC*^ function *in vivo* is proteolytic separation of Hh from its cholesteroylated GFP tag, as demonstrated in our work (Figure 5). Such proteolytic processing is not restricted to GFP tagged proteins, because we also found differently sized Shh variant isoforms in Scube2-containing cell culture media *in vitro*, with the two smallest forms lacking their C-terminal tags and corresponding in size to untagged soluble Shh. These forms originated from a larger precursor present in the corresponding cellular fractions (Figure 7). We conclude from these results that lipidated Hh and Shh proteins are prone to proteolytic C-terminal peptide processing, and that—especially if combined with retained biofunction of non-cholesteroylated HhN variants—Hh cholesteroylation is not likely involved in Hh relay and the final step of Ptc receptor binding at the receiving cell surface. This possibility is supported by comparable binding affinities of Shh and ShhN toward Ptc (Pepinsky et al., 1998; Burke et al., 1999) and by the observation that Shh proteins released by Scube2 (a molecule that acts upstream of Ptc) always become devoid of their C-terminal cholesteroylated peptide tags (Jakobs et al., 2014). Notably, replacing the Hh cholesterol with CD2 or the glycosyl-phosphatidylinositol (GPI)-anchoring signal of *Drosophila* Fascilin I that also tether Hhs to the cell membrane of producing cells—but are resistant to proteolytic processing at the cell surface—abolishes all Hh activity in *Drosophila* wing discs (Strigini and Cohen, 1997; Burke et al., 1999; Gonzalez-Mendez et al., 2017). This suggests that the specific purpose of Hh C-terminal peptide cholesteroylation is to tether Hhs to the cell surface only until their proteolytic removal is required. Hh would then separate from the lipid (or the lipidated tags in our experiments) as a prerequisite for its unimpaired binding to its receptor Ptc at the surface of receiving cells (Gong et al., 2018). Along this line, we suggest that variably impaired signaling of Hh variants with small C-terminal tags, as observed in our study, may be caused by changed spatiotemporal Hh release and increased unregulated proteolysis (e.g., unregulated background “leakage” from the cell surface). Indeed, a general feature of sheddase recognition is that substrates require a susceptible membrane-proximal stalk of sufficient length between the membrane and the proximal extracellular globular domain to permit access of the protease to substrate (Baran et al., 2013). Thus, membrane proteases cannot process domains if they are too close to the surface or even at the surface, but they are made accessible by CUB-domain dependent substrate activators such as Scube2 or procollagen C-proteinase enhancers 1 and 2 (PCPE1/2) to induce spatiotemporally controlled shedding (Takahara et al., 1994; Blanc et al., 2007; Jakobs et al., 2017). In the opposite scenario, insertion of tags may expose sheddase cleavage sites to increase their unregulated proteolytic processing and leakage from the cell (in a protein with extended terminal lipidated peptides, as shown for Shh^*HA*^, ^*HA*^Shh^*HA*^, and Shh^*Strep*^; Figure 7; Jakobs et al., 2017). Our observation of unregulated Hh^*HA*^ and Hh^*Strep*^ background release from their expressing cells, but no unregulated release of Hh^Δ*HA*^ representing the most bioactive variant supports this possibility.

### Hh Cholesteroylation and Direct Protein–Protein Contacts Are Required for Hh Multimerization *in vivo*

Another notable finding of our work addressed the long-standing observation that non-palmitoylated ^*C*85S^Hh suppresses endogenous Hh biofunction during wing development (Figure 3; Lee et al., 2001). Our data indicate that suppressed endogenous Hh activity by ^*C*85S^Hh is not a direct consequence of palmitate loss. In contrast, we suggest a scenario in which inactive ^*C*85S^Hh interacts with endogenous Hh at the producing cell surface, in turn diluting bioactive Hh concentrations down and reducing the overall signaling activity (Schürmann et al., 2018). These interactions are C-cholesterol-dependent, because ^*C*85S^HhN lacking the lipid did not suppress endogenous Hh biofunction. We also reveal that sequence or length of the cholesteroylated peptide affects the suppression of endogenous Hh biofunction caused by the C85S mutation or the N-terminal HA tag, either by sterically impaired interactions with endogenous Hh clusters—a possible direct effect of the tag—or more likely by increased unregulated processing of the tagged peptide—an indirect effect of the tag that would lead to the loss of the cholesteroylated peptide and thus reduce cholesterol-dependent Hh interactions at the cell surface. Besides the cholesteroylated peptide, another known facilitator of Hh clustering is HS (Vyas et al., 2008), the level of sulfation and differential subcellular distribution of which have been established to regulate the assembly of the Wnt family member Wnt8 into punctate structures at the cell boundary and inside cells (Mii et al., 2017). Given the possibility that HS likewise regulates the association and internalization of Hhs, impaired HS interactions as a consequence of the insertion of the Strep tag, as indicated by our study (Figure 6), may thus have contributed to changed Hh bioactivities and altered dominant-negative properties *in vivo*, yet only to a very limited degree.

Taken together, our work demonstrates that modified C-terminal cholesteroylated Hh peptides can reduce the relative level of Hh multimers *in vitro* or *in vivo*, depending on the sequence, size, and site of insertion of the tag. This observation suggests that Hh multimerization during development is not a mere consequence of Hh lipidation, as previously assumed, but strongly influenced by the associated C-terminal Hh peptide. Therefore, both the C-terminal peptide and the lipid are important decisive factors for Hh biofunction at the surface of Hh producing cells. As a possible explanation, our work also demonstrates that C-terminal Hh peptides are prone to proteolytic processing, which would release the morphogen from the producing cell surface or from lipophilic carriers, such as cytonemes, present in the Hh receiving compartment. This, in turn, suggests that the final step of Ptc receptor binding does not depend on the Hh-linked lipid, consistent with the observed role of Scube2 to increase proteolysis of both lipidated Shh peptides as a prerequisite for its release from the producing cell surface (Jakobs et al., 2014), and also consistent with the ability of the artificially produced, non-cholesteroylated Hh protein to signal *in vitro* (Zeng et al., 2001; Dawber et al., 2005) and *in vivo* (Porter et al., 1996a; Lewis et al., 2001; Li et al., 2006).

## Data Availability Statement

The original contributions presented in the study are included in the article/Supplementary Material, further inquiries can be directed to the corresponding author/s.

## Author Contributions

DM: investigation, methodology, visualization, formal analysis, writing—original draft preparation, writing—reviewing, and editing. PK and PJ: investigation and formal analysis. SS and KE: investigation. GS: investigation, methodology, and writing—reviewing. KG: conceptualization, supervision, writing—reviewing, and editing, funding acquisition. All authors contributed to the article and approved the submitted version.

## Conflict of Interest

The authors declare that the research was conducted in the absence of any commercial or financial relationships that could be construed as a potential conflict of interest.
