# Supplementary material for: C-Terminal Peptide Modifications Reveal Direct and Indirect Roles of Hedgehog Morphogen Cholesteroylation
Source: Front Cell Dev Biol. 2021 Jan 12;8:615698. doi: 10.3389/fcell.2020.615698 (PMC7835520; doi:10.3389/fcell.2020.615698)
Supplement: Supplementary file 1 [file Data_Sheet_1.docx]

Supplementary Material

#
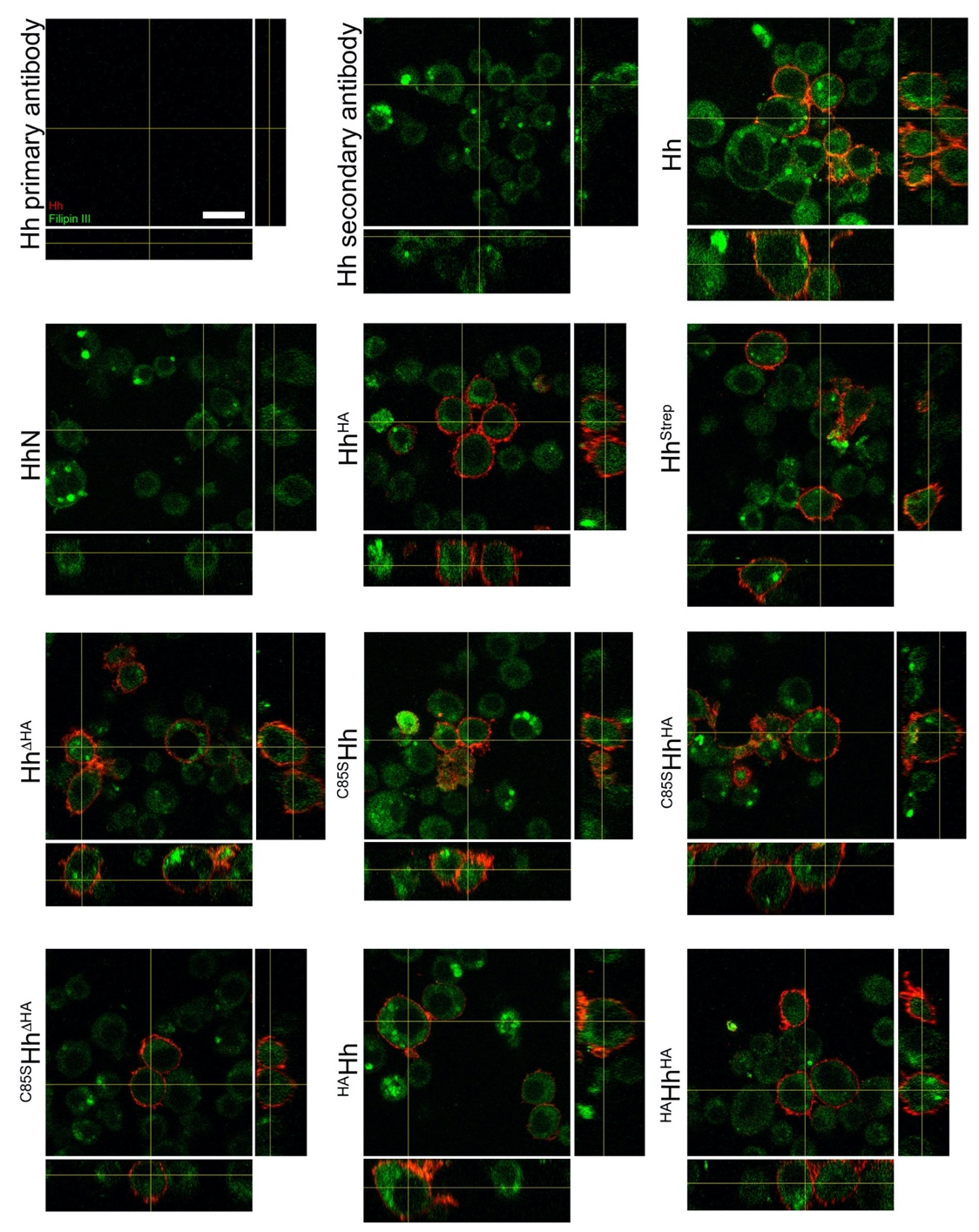


**Figure S1: Confocal microscopy analysis of *Drosophila* S2 cells transfected with hh and C-terminally modified hh variants.** Cells were stained with anti-Hh antibodies and subsequently labeled with Cy3 conjugated anti-rabbit IgG antibodies (red). Filipin III counterstain was used to visualize the cell membranes (green). Primary or secondary antibody only controls were used to rule out any unspecific staining. Hh and all C-terminally modified Hh variants were correctly expressed and secreted to the cell surface. In contrast, HhN, which represents the non-lipidated form of Hh, was not detected at the cell surface (due to its unimpaired secretion caused by the deletion of the C-terminal cholesterol anchor). Orthogonal views of one Z-stack per condition are shown. Scale bar 10µm.

**
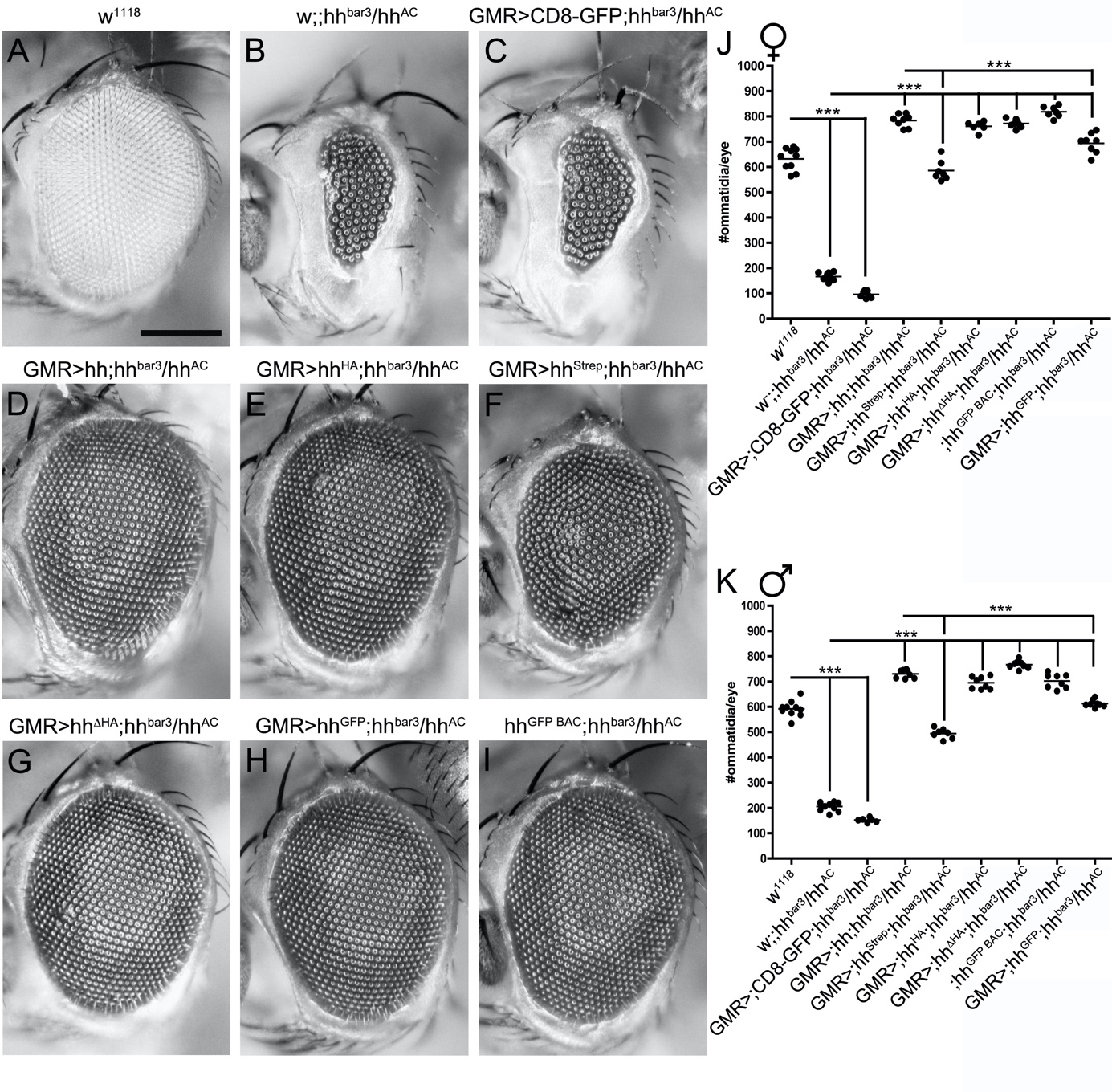
**

**Figure S2: C-terminal Hh modifications only mildly affect short-range signaling in the *Drosophila* eye at 25°C, demonstrating protein folding and signaling in this system.** A) Wild-type fly eyes consist of several hundreds of ommatidia that develop in response to Hh signaling. Scale bar 100 µm. B) In hh^bar3^/hh^AC^ female flies, eye development was strongly impaired. C) CD8-GFP expressed in hh^bar3^/hh^AC^ flies under eye disc-specific GMR control did not rescue the hh^bar3^/hh^AC^ loss-of-function phenotype, similar to what we observed at 18°C. D) Expression of untagged Hh and E-I) C-terminally modified Hh variants fully rescued eye development in hh^bar3^/hh^AC^ flies to wild-type level or even above, with the exception of Hh^Strep^. J,K) Quantification of A-I in female and male flies, respectively. Statistical significance was determined by one-way ANOVA followed by Bonferroni’s multiple comparison test. ***P<0.001. All crossings were carried out at 25°C.

**
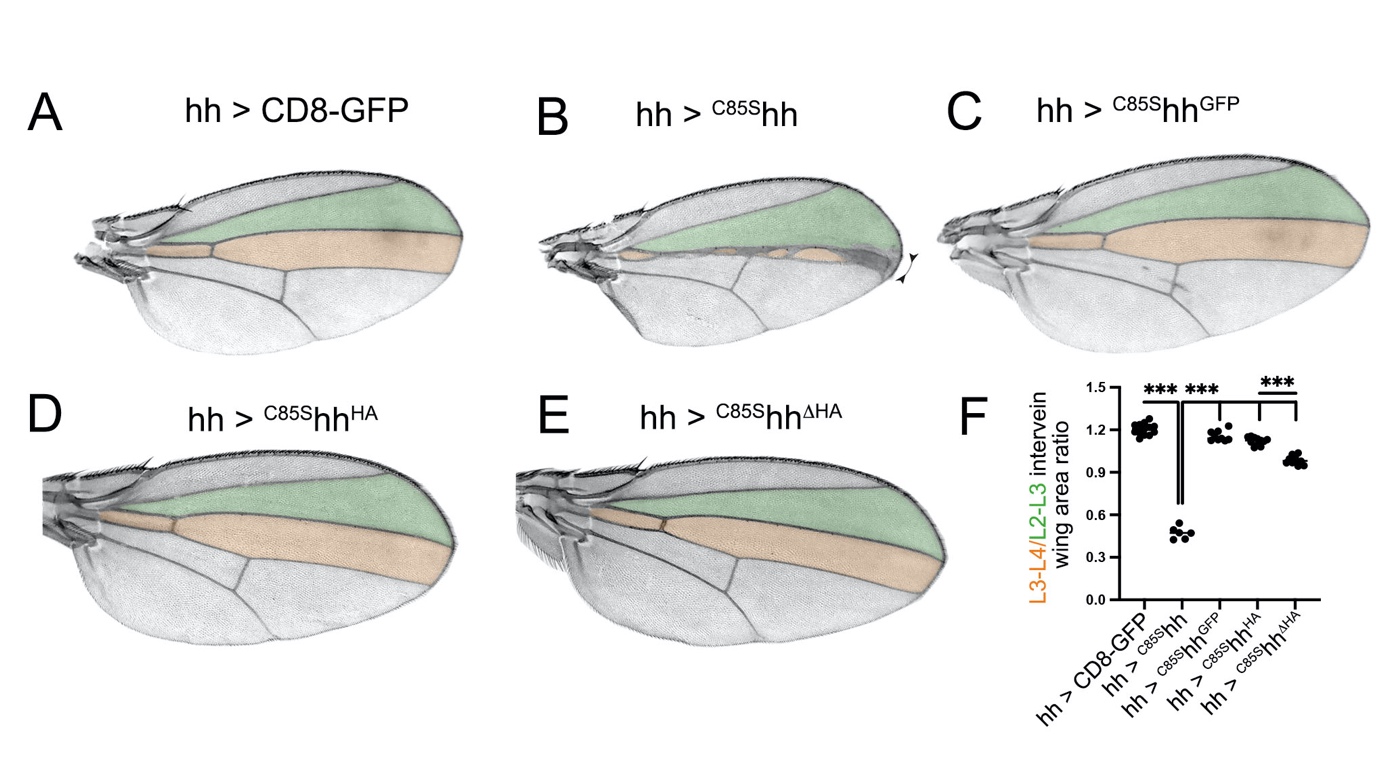
**

**Figure S3: C-terminal Hh modifications attenuate dominant-negative activities of non-palmitoylated ^C85S^Hh variants in the *Drosophila* wing at 25°C.** A-E) Wing patterning as a consequence of Hh-Gal4-driven overexpression of non-palmitoylated Hh variants. F) Quantification of A-E. Statistical significance was determined by one-way ANOVA followed by Sidak’s multiple comparison test. ***P<0.001.

**
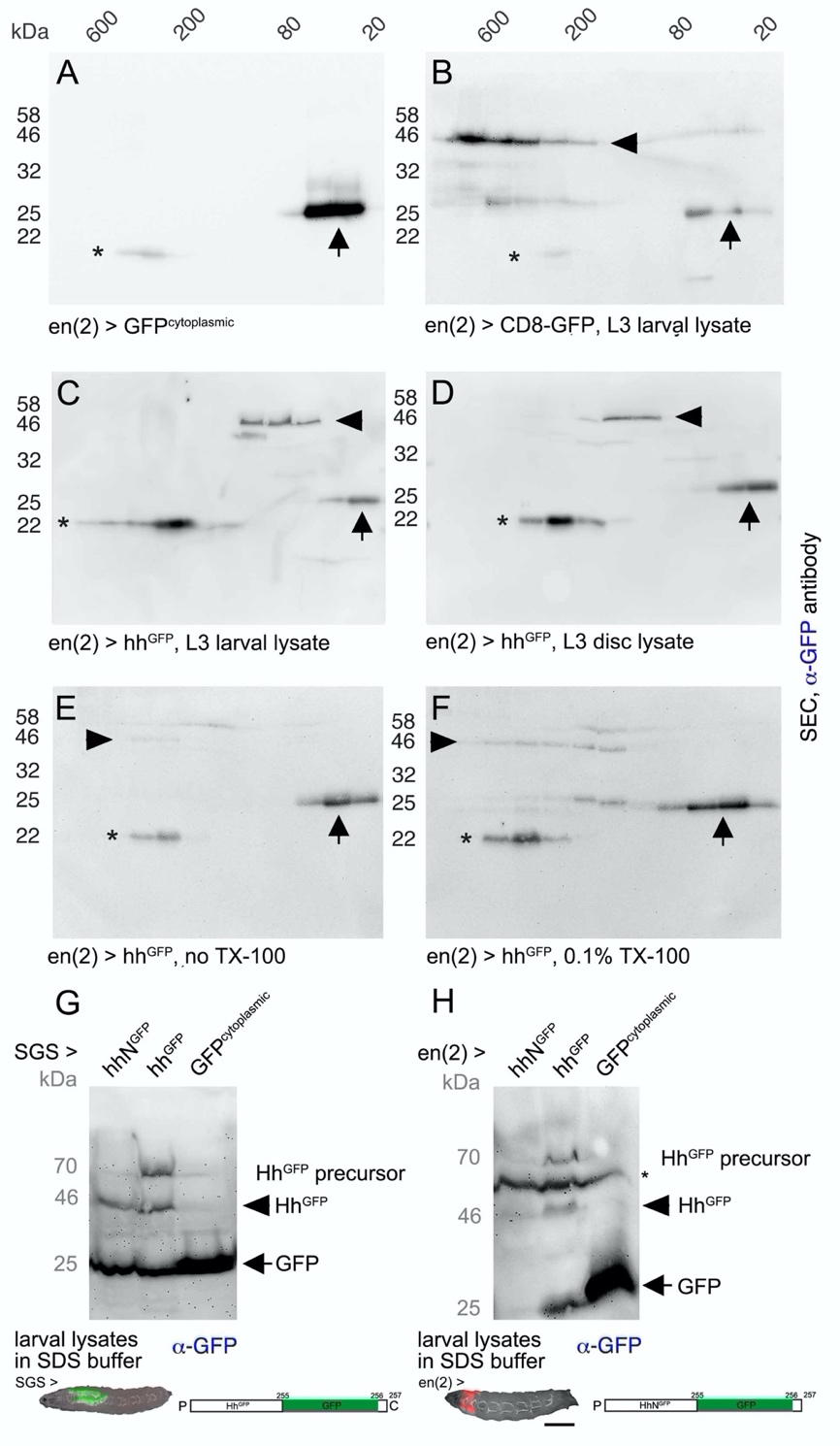
**

**Figure S4: Hh^GFP^ proteins undergo C-terminal processing in larval disc tissue and in salvary glands.** A-C) SEC of larval homogenates expressing A) GFP^cytoplasmic^, B) CD8-GFP or C) Hh^GFP^ stained with α-GFP antibodies (Rockland) confirmed C-terminal processing of Hh^GFP^ fusion proteins. Arrows highlight monomeric processed GFP, arrowheads full-length fusion proteins and asterisks unspecific staining. D) Larval imaginal discs were dissected, homogenized and subjected to SEC followed by immunoblotting. Similar to whole larval homogenates, processed 27 kDa GFP was detected in disc tissue (arrow). E-F) SEC of larval homogenates either with (E) or without (F) Triton X-100 to distinguish between soluble (E) or insoluble (F) Hh^GFP^ or GFP. G-H) To rule out that GFP cleavage occurred during SEC, we directly immunoblotted homogenates of larvae expressing Hh^GFP^ either in salvary glands (SGS>) or in disc tissues [en(2)>] and confirmed the presence of abundant processed GFP (arrows).

**
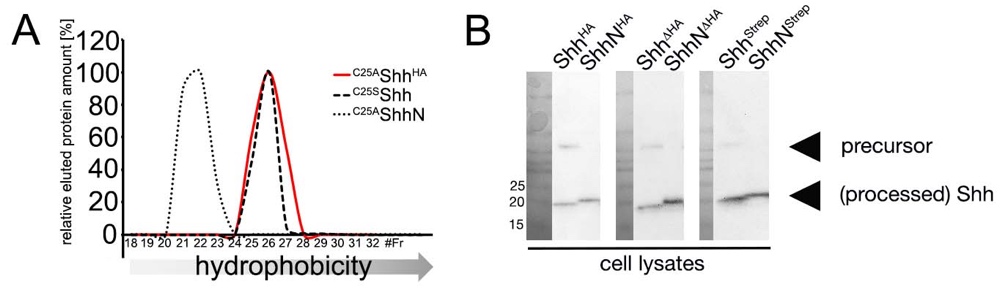
**

**Figure S5: Reverse-phase HPLC and immunoblotting confirmed complete cholesteroylation of C-terminally tagged** **Shh mutants.** A) Non-lipidated soluble control ^C25A^ShhN, cholesteroylated ^C25S^Shh and ^C25A^Shh^HA^ were produced in Bosc23 cells. All proteins bound to and eluted from a hydrophobic C4 column, but soluble, non-lipidated ^C25S^ShhN was less hydrophobic than ^C25S^Shh and ^C25A^Shh^HA^ isolated from Bosc23 cell lysates. This confirmed unimpaired cholesteroylation of HA tagged ^C25A^Shh^HA^. Elution profiles were adjusted relative to the highest protein amount in a given fraction (set to 100%). Fr#: fraction number. B) Immunoblots of cell lysates also confirmed unimpaired cholesteroylation of all C-terminally tagged Shh variants: The 46 kDa precursor is autocatalytically processed into cholesteroylated 20 kDa proteins (Shh^HA^, Shh^ΔHA^ and Shh^Strep^) and lipidation causes increased electrophoretic mobility compared to non-cholesteroylated ShhN variants (compare Shh^*^ with corresponding ShhN^*^) as described previously (Pepinsky et al., 1998; Porter et al., 1996).

Pepinsky, R.B., Zeng, C., Wen, D., Rayhorn, P., Baker, D.P., Williams, K.P., Bixler, S.A., Ambrose, C.M., Garber, E.A., Miatkowski, K., Taylor, F.R., Wang, E.A., Galdes, A., 1998. Identification of a palmitic acid-modified form of human Sonic hedgehog. J Biol Chem 273, 14037-14045.

Porter, J.A., Young, K.E., Beachy, P.A., 1996. Cholesterol modification of hedgehog signaling proteins in animal development. Science 274, 255-259.


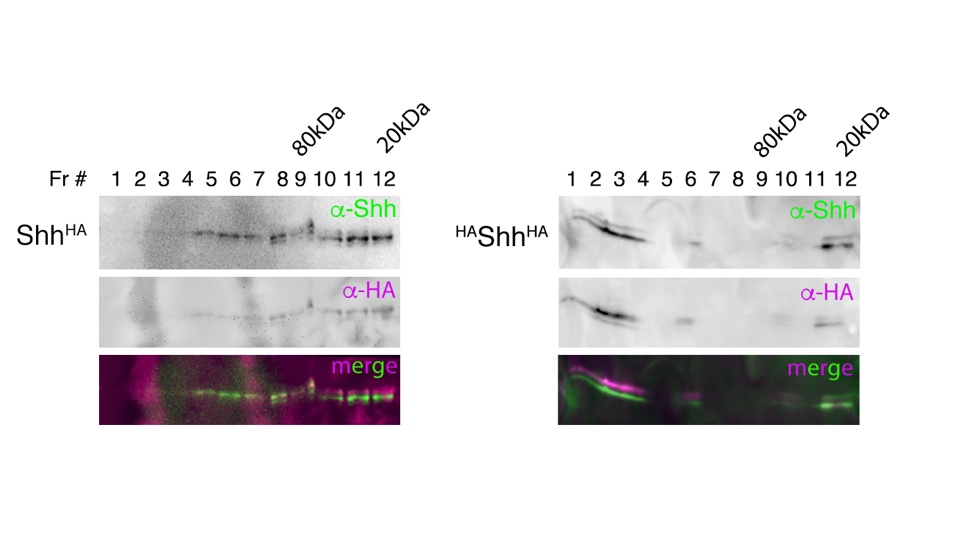


**Figure S6: Loss of C-terminal hemagglutinin (HA) tags in monomeric Shh.** SEC of HA tagged soluble Shh secreted from Bosc23 cells. Fractions 1-8 (>80kDa) denote multimeric Shh and fractions and 9-12 (< 80 kDa) monomeric Shh. Polyclonal α-Shh antibodies (colored green in the inverted, false-colored merged blot) detected multimeric and monomeric Shh^HA^ and ^HA^Shh^HA^ proteins. ^HA^Shh^HA^ is dually tagged, similar to ^HA^Hh^HA^ (Figure 4). Monoclonal α -HA antibodies, however, detect multimeric Shh^HA^ and ^HA^Shh^HA^, but does not bind the monomeric forms (magenta in the inverted, false-colored merged blot). This suggests Shh multimer disintegration as a consequence of terminal peptide processing at the surface of the producing cell, or that proteolytic processing of terminal lipidated peptides prior to their solubilization prevented this process.
